# Contextualising video game engagement and addiction in mental health: the mediating roles of coping and social support

**DOI:** 10.1016/j.heliyon.2020.e05340

**Published:** 2020-11-16

**Authors:** Clara E. Moge, Daniela M. Romano

**Affiliations:** a Department of Psychology and Language Sciences, University College London, London, WC1H 0AP, United Kingdom; b Department of Information Science, University College London, London, WC1E 6BT, United Kingdom

**Keywords:** Psychology, Video game addiction, Engagement, Mental health, Coping, Perceived social support

## Abstract

**Introduction:**

A challenge in defining Internet Gaming Disorder (IGD) is discriminating pathological gameplay from an excessive, yet benign, involvement in video games. Although previous research has explored this theoretical distinction in the context of general computing activities, it merits consideration with regards to online gaming. Additionally, whilst comorbidities of addicted gaming and mental health outcomes have been robustly demonstrated, few studies have examined the role of mediating factors that may contextualise this relationship. As such, the present study aims to validate the distinction between addiction and engagement in online gaming, by considering the mediating roles of coping and social online and offline support in mental health.

**Method:**

One hundred and thirty-five participants completed the Computer Engagement/Addiction Questionnaire (CEAS), Depression-Anxiety-Stress Scale (DASS-21), Brief Approach-Avoidance Coping Questionnaire (BACQ) and two versions of the Multidimensional Scale of Perceived Social Support (MSPSS).

**Results:**

Correlational analyses showed a clear distinction between gaming addiction and engagement in the context of all of depression, stress and in particular anxiety (DAS) not found in previous studies. Multiple mediation analysis showed a significant mediating effect of coping, (specifically withdrawal/resignation coping) on the relationship between video game addiction and symptoms of DAS. Offline perceived social support was a significant partial mediator in the relationship between gaming addiction and depression, as compared to any kind of online social support. The results support the distinction of the addiction and engagement concepts in gaming. This study may inform future clinical classifications of IGD, with implications on how pathological gaming is treated.

## 1 Introduction

Online video games are a maturing, contemporary medium for entertainment gaining prominence across the popular culture landscape (Muriel and Crawford, 2018). Due to their rapid absorption amongst adolescents, increasing research has investigated the possibility of harmful, pathological involvement in this recreational pastime (Nuyens et al., 2019). The classification of Internet Gaming Disorder (IGD) as a mental health disorder in the International Classification of Diseases (ICD-11; World Health Organisation, 2018) synthesises some of these insights, identifying individuals who (1) lack control over their gaming behaviour, (2) prioritize gaming over other activities and (3) persist with this pattern despite adverse consequences. For a positive diagnosis, individuals must fulfil all three of these criteria, with associated behaviours being evident for at least 12 months (World Health Organisation, 2018).

Despite some recognition in psychiatric nomenclature, IGD is noted in the Diagnostic and Statistical Manual of Mental Disorders (DSM-5) as a tentative disorder meriting further research (American Psychiatric Association, 2013). Referents of video game overuse have also remained inconsistent, with terms like ‘problematic’, ‘compulsive’, ‘dependent, and ‘pathological’ gaming being used interchangeably. Perhaps most controversially has been the informal categorisation of problematic gaming as a technological ‘addiction’. Colloquially, the addiction narrative is recurrent in discourse amongst problematic gamers themselves (Chappell et al., 2006) and academics (Bean et al., 2017). Although excessive gaming is less obviously associated with a potential for unintended harm than other addictions (Seah and Cairns, 2008), it shares characteristics like diminished control, preoccupation, tolerance and withdrawal (Saunders et al., 2017). These behavioural and psychological similarities between pathological gambling and gaming have led to the examination of the latter within frameworks of addiction (Kuss and Griffiths, 2012). However, no academic consensus has been reached regarding the labelling of excessive Internet gaming as an addiction (Ferguson and Colwell, 2020). Aside from constituting a barrier to future research, this also raises concerns related to the stigma associated with social labelling of gaming ‘addicts’.

### 1.1 Video game addiction and engagement

A major challenge in defining IGD is discriminating pathological from excessive gameplay. The latter is benign involvement in video games, and key to the success of the game, while the former is connected to mental health.

Introduced by Charlton and Birkett (1995), the notion of high computer engagement characterises individuals with high computer usage that do not suffer negative psycho-social ramifications. Drawing from Brown (1991) addiction framework, Charlton (2002) put forward the idea that excessive involvement with computing systems may be dissociated into discrete engagement and addiction components. Through factor analysis, he found evidence of a clear ‘addiction’ factor significantly loading onto four of Brown's criteria (labelled ‘core’ criteria) for behavioural addiction.

More recently, findings from confirmatory factor analysis have supported the hypothesis of addiction and engagement as separate constructs with regards to gaming (Deleuze et al., 2018). Whilst Griffiths (2010) argues that highly engaged and addicted gaming cannot be discriminated on the basis of time spent playing, some studies have shown they *can* be distinguished based on the occurrence of negative ‘health complaints’ (Brunborg et al., 2013); and more severe symptoms of psychopathology (Loton et al., 2016; Krossbakken et al., 2018).

Although assessment methods differ between studies, these findings have fuelled the idea that the manifestation of negative psychosocial health outcomes may be a useful axis of comparison between engagement and addiction. This is why the Computer Engagement/Addiction Scale (CEAS; Charlton and Danforth, 2007) may have an advantage over tools like the Gaming Addiction Scale (GAS; Lemmens et al., 2009) and IGD-9SF (Pontes and Griffiths, 2015), because if used in conjunction with mental health assessments it may draw an important line between excessive (highly engaged) and pathological (addicted) players.

### 1.2 The comorbidity between Internet gaming and reduced mental health

Assessing functional impairment outside of the in-game context, research has previously focused on the effect of IGD on social relationships (Allison et al., 2006), vocational and educational outcomes (Jeong and Kim, 2011), and physical and psychological health (Ferguson et al., 2011). Symptoms of depression and anxiety have emerged cross-culturally as significant correlates of high video game dependency (Mentzoni et al., 2011; Stockdale and Coyne, 2018; Gentile et al., 2011). Recent research has suggested this relationship between conditions of psychopathology and gaming addiction may be bi-directional (Liu et al., 2018). Overall, these findings run in parallel with the literature showing an association between depression and other addiction disorders (Morozova et al., 2015; Neupane, 2016), and more specifically between depression and Internet addiction (Brunborg et al., 2014).

Whilst the aforementioned research supports the conclusion that pathological gaming increases the likelihood of poor psychological health outcomes, the intrinsic difference between video game addiction and engagement (Charlton and Danforth, 2007; Deleuze et al., 2018) suggests engagement should not compromise mental health. However, there remains little evidence indicating this is the case (Loton et al., 2016). The lack of such a distinction may explain inconsistencies in results reporting the relationship between ‘excessive’ gaming and psychopathology (e.g. King et al., 2011). In addition, there is a need for future research samples to include females, as although the proportion of female gamers is increasing, most research still uses all-male samples (Lopez-Fernandez et al., 2019). Samples should also be broadened to include adults in addition to adolescents in studies of addicted gaming (Seok and DaCosta, 2014).

### 1.3 The mediating role of coping

Coping may be an external variable which contextualises the relationship between addictive gaming behaviour and reduced mental health. Coping is defined as psychological and behavioural efforts employed to reduce stress caused by external or internal events appraised as physically or mentally demanding (Folkman et al., 1986). Approach and avoidance are two broad dimensions of coping which reflect an orientation either towards or away from a stressor (Roth and Cohen, 1986). Approach coping has generally been established as an adaptive strategy to manage stressors and is inversely associated with poor mental health (Thompson et al., 2016). In contrast, avoidance coping is achieved through withdrawal or resignation, and diversion (Finset et al., 2002), and is often viewed as a temporary, short-term measure to prevent emotional consequences of stress (Sweeny et al., 2016). In the long term, the use of avoidant strategies to regulate negative emotionality is generally considered maladaptive (Sirois and Kitner, 2015).

In previous research, avoidance coping has been identified as a significant mediator between symptoms of psychopathology, specifically depression and anxiety, and Internet Addiction (IA; Brand et al., 2014; Cheng et al., 2015; McNicol and Thorsteinsson, 2017). The tendency to deploy maladaptive (avoidant) more than adaptive (active) coping strategies has been found in clinically diagnosed male Internet addicts (Senormanci et al., 2014), and independent of sex in individuals with substance use issues (Lee-Winn et al., 2018) and behavioural addictions like gambling (Jauregui et al., 2017). Consistent with the finding that excessive Internet use has described as a coping strategy in itself (Tang et al., 2014), Plante et al. (2019) found that using online games to avoid stress predicts symptoms of gaming addiction. This points to the conception of gaming as a means of escape from reality for addicted gamers (Beranuy et al., 2013). In line with these claims, a recent study by Schneider, King and Delfabbro (2017) found that adolescents using cognitive and behavioural avoidance coping strategies were at significantly greater risk of developing IGD symptoms. Avoidance coping thus emerges as a salient psychological function and negative outcome of pathological gaming behaviour.

However, there are some gaps in the existing research. First, emerging insights from longitudinal research suggest poor psychological health and wellbeing is an outcome, rather than a predictor, of pathological gaming and Internet use (Gentile et al., 2011; Wartberg et al., 2019; Coyne et al., 2020). This should be considered in future cross-sectional studies, given that the majority of those to date hypothesise an opposite directionality (Herrero Olaizola et al., 2019). Secondly, whilst research has shown avoidance coping is implicated in the maintenance of IA, this does not necessarily mean that approach coping is a significant contributor in reduction or avoidance of IA given that avoidance and approach coping do not represent bipolar ends on a spectrum (Finset et al., 2002). Therefore, it is important for emerging research to consider both constructs as opposed to avoidance coping on its own.

Taken together, research into approach/avoidance coping in IA and IGD, including research pointing towards the co-occurrence of psychological distress with these conditions, concretises the relevance of coping in the study of gaming addiction. To date, only Loton et al. (2016) have investigated the relationship between video game addiction and engagement, coping, and mental health. Through multiple mediation analysis, they found a direct effect of video game addiction on depression, stress and anxiety, and a lack thereof between engagement and depression and stress. This is partial empirical support for the hypothesis that engagement does not lead to any adverse psychological health consequences. Loton et al. identified maladaptive coping as a significant but partial mediator of the relationship between gaming addiction and depression, anxiety, and stress. This evidence favours the inclusion of coping as a mediator in future quantitative models of video game addiction. However, as coping strategy was not a full mediator of the video game addiction-mental health relationship, further research should aim to identify additional variables that may provide a more complete explanation of this relationship.

### 1.4 The potential mediating role of social support

One contextual factor which may be responsible for the remaining variability in the relationship between video game addiction and reduced mental health is social support. As conveyed in its definition, social attachments are significant and positive contributors to the maintenance of psychological health (House, 1983). Individuals' subjective evaluations of their social relationships (perceived social support) has been emphasised as a significant predictor of positive health outcomes, whilst objective measures (received social support) have shown less consistency (Reinhardt et al., 2006).

There are multiple reasons why the perceived availability of social support is relevant to include in quantitative models of the gaming addiction-mental health relationship. Firstly, empirical descriptions of IA have emphasised the co-occurrence of difficulties in social contexts (Young, 2007). This is illustrated in the systematic finding of a negative dose-effect relationship between IA and self-reported social support (Gunuc and Dogan, 2013; Wu et al., 2016), suggesting individuals who exhibit problematic Internet use behaviours are also more likely to be socially isolated. Longitudinal studies have furthered this by showing that long-term Internet use specifically to play online video games leads to decreases in offline perceived social support (Blais et al., 2008). Secondly, alongside coping, social support is an important component in clinical interventions for addictive disorders (Petry and Weiss, 2009; Kim et al., 2017), and generally for both physical and mental health (Uchino, 2009; Taylor and Stanton, 2007). Thirdly, there is a need for research to consider the contribution of social support in the transpiration of pathological gaming whilst making the delineation between addiction and engagement (Seok and DaCosta, 2014). For these reasons, perceived social support (PSS) may mediate the relationship between addicted Internet gaming and reduced mental health.

Curiously, the potential mental health benefits of *online* social support for excessive gamers has emerged as a controversial topic, underlain by mixed findings. Consistent with the ‘lonely gamer’ stereotype which portrays young adult males with few offline social ties (Vermeulen and Van Looy, 2016), the displacement hypothesis argues that the social world accessed through the Internet replaces higher quality, real-life social contact with others (Kraut et al., 1998; Blais et al., 2008; Miyata and Kobayashi, 2008; Gentile et al., 2011). An opposite school of thought, the augmentation hypothesis (Katz and Aspden, 1997), proposes that the Internet allows excessive gamers to strengthen social ties with others. For example, Kraut and Burke (2015) reported that using the Internet to communicate with strong ties was associated with higher perceptions of social support, and lowered symptoms of depression. The potential dualistic effects of displacement and augmentation suggest a more complex picture of excessive gaming, in which (1) social support received offline may decrease for addicted gamers, but (2) social support received online may increase and adequately support psychological well-being (Seok and DaCosta, 2014). Therefore, there is a need for research to consider the distinction between social relationships maintained on and offline.

## 2 The present study

Whilst past research has established a negative relationship between problematic gaming and poor mental health, there has been little consideration of the addiction/engagement dissociation. In addition, no study to our knowledge has shown whether addiction and engagement are fully dissociable in the context of depression, anxiety and stress. This is important to inform the development of meaningful clinical assessment criteria and treatment strategies for IGD, and to avoid conflation of benign gaming with pathological gaming. As such, the first aim of the present study is to validate the distinction between addiction and engagement in the context of psychological health outcomes.

H_1_ There is a positive correlation between video game addiction, but not engagement, and depression, anxiety and stress (DAS; see Figure 1.1).

**Figure 1:**
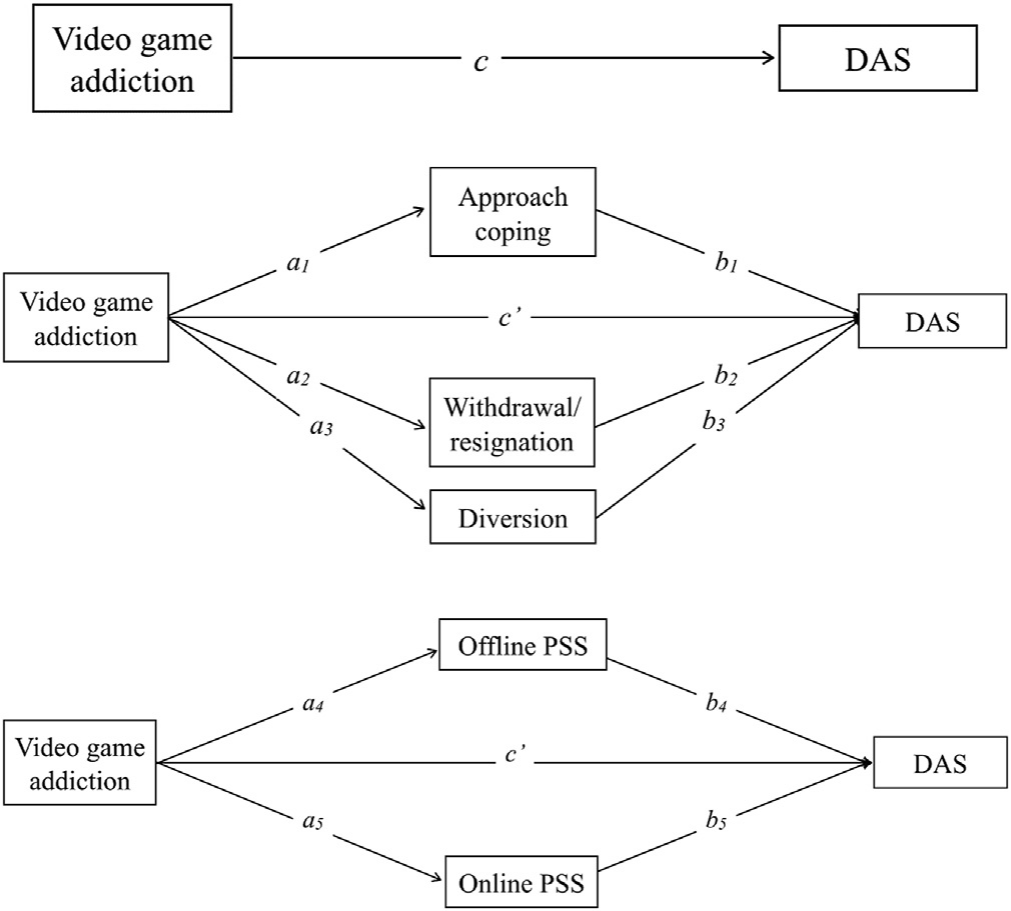
Figure 1 1. First diagram, causal path diagram showing the total effect (correlation) of video game addiction on symptoms of DAS, as anticipated by H_1_. *Note*. Depression, anxiety, and stress will be examined as psychometrically distinct constructs. Second diagram, mediation model of video game addiction on symptoms of DAS, with coping as a mediator. Pathways *a* and *b* represent the indirect effect, whilst *c’* represents the direct effect of video game addiction on DAS when coping mediators are held constant. Third diagram, mediation model of video game addiction on symptoms of DAS, with PSS as a mediator. Pathways *a* and *b* represent the indirect effect, whilst *c’* represents the direct effect of video game addiction on DAS when PSS mediators are held constant.

A second aim of the current research is to validate the role of coping as a mediator in the relationship between video game addiction and symptoms of DAS, which has not received much support (Loton et al., 2016).

H_2a_ Video game addiction has an indirect effect on DAS through high avoidance coping (withdrawal/resignation and diversion) and low approach coping (see Figure 1.2).

H_2b_ Video game engagement will not have any effect on DAS, except through high avoidance and low approach coping.

Lastly, this research seeks to examine the role of perceived social support, PSS, as another mediator between addiction and mental health, distinguishing between online and offline forms of support. Offline PSS plays a significant role in the maintenance of gaming addiction, so it is expected to contribute significantly to reduced mental health. However, given the lack of research in this area, no specific predictions are made regarding the role of online support for both addicted and engaged gamers. Overall, defining the potential mediating roles of coping and PSS in IGD is important to guide clinical treatment design.

H_3a_ Video game addiction has an indirect effect on DAS through low PSS, especially offline support (see Figure 1.3).

H_3b_ Video game engagement will not have an indirect effect on DAS, except through low PSS, especially for offline support.

## 3 Method

### 3.1 Participants

In total, 217 participants were recruited through volunteer sampling via word-of-mouth, social media (e.g. Reddit), and campus advertising. From those, 77 participants were excluded based on incomplete data, and a further 5 participants were removed due to appearing spurious or lacking fluency in English on demographic measures. The final sample consisted of 135 participants ranging from ages 18 to 47 (*M*_*age*_ 24.29 = years, *SD* = 6.48 years). Of those, 82 participants identified as male, 47 as female and 6 as other or non-binary. The majority of participants declared American nationality (42.6%), with the rest being from European countries (22.7%), British (12.7%), and smaller cohorts from other countries across Asia and Africa (22.0%). The estimated time spent playing video games weekly (*M*_*time*_ = 20.28 h, *SD* = 15.52 h) ranged from 0 to 70 h per week.

The inclusion criteria required participants to be over 18 years old. Ethical approval for the study was granted by UCL Department of Information Studies Research Ethics Committee.

### 3.2 Materials

#### 3.2.1 Computer Engagement/Addiction Scale (CEAS)

The Computer Engagement/Addiction Scale (Charlton and Danforth, 2007) is a 24-item scale measuring two types of engagement with online video games; dysfunctional (Addiction subscale) and healthy engagement (Engagement subscale). Responses are given on a seven-point Likert scale ranging from ‘completely disagree’ to ‘completely agree’, coded such that higher scores indicate greater addiction and engagement. In the present study, the scale was modified such that the game-specific term ‘Asheron's Call’ was substituted with the more general term ‘video games’ (as in Loton et al., 2016). Both subscales of the CEAS demonstrated a good reliability in this study (Cronbach's α = .80 for Addiction and α = .86 for Engagement).

#### 3.2.2 Depression-Anxiety-Stress Scale (DASS-21)

The DASS-21 is an established short-form, self-report measure of mental health symptoms, with good reliability and validity reported cross-culturally (Lovibond and Lovibond, 1995). The scale is composed of three 7-item subscales, measuring depression, anxiety, and stress. Agreement with each statement is recorded on a 4-point Likert scale ranging from ‘0 = Did not apply to me at all’ to ‘3 = Applied to me very much, or most of the time’. In the present study, the Depression subscale demonstrated excellent reliability (α = .91), the Anxiety subscale showed acceptable reliability (α = .77) and the Stress subscale showed good reliability (α = .84).

#### 3.2.3 Brief approach/avoidance coping questionnaire (BACQ)

The BACQ (Finset et al., 2002) is a 12-item questionnaire consisting of two subscales measuring approach and avoidance coping strategies. Agreement with each statement is given on a 5-point Likert scale ranging from ‘completely disagree’ to ‘completely agree’. Similar to Loton et al. (2016), each subscale in the present study had questionable reliability (α = .61 for Approach, α = .61 for Resignation/Withdrawal, α = .62 for Diversion). Billings and Moos (1981) suggest employing singular coping strategies may relieve stress without the need for other strategies to be deployed. As such, reliability tests are limited in their applicability for assessing the psychometric robustness of coping measures like the BACQ. Given Cronbach's alpha coefficients are affected by the number of items in a scale, it is also possible that separating avoidance coping into two 3-item subscales leads to a low internal consistency. All coefficients nonetheless exceeded the proposed minimum required for research (α = .60; Nunnally and Bernstein, 1995), and were included in the statistical analysis.

#### 3.2.4 Multidimensional Scale of Perceived Social Support (MSPSS)

The MSPSS is a measure of perceived social support (Zimet et al., 1988). The questionnaire contains 12-items, 4 items each measuring PSS from three sources: friends, family, and significant others. Each item is measured on a 5-point Likert scale, ranging from ‘Very strongly disagree’ to ‘Very strongly agree’.

Two versions of the MSPSS are presented in this study; the original was used to measure PSS in offline contexts (e.g. support from people who were first encountered in-person), and a modified version was used to measure PSS online (e.g. social support which is received via phone calls, text messaging and the Internet). The wording of the statements for each item was rephrased to emphasise the specificity of the online context (see Appendix A). For both versions, all subscales showed excellent reliability (offline MSPSS scale: α = .95 for Friends, α = .92 for Family and α = .96 for Significant Other; online MSPSS scale: α = .95 for Friends, α = .91 for Family and α = .97 for Significant Other).

### 3.3 Procedure

On the web-based experimental tool Gorilla (www.gorilla.sc), participants provided their informed consent and filled out a demographics questionnaire (age, gender, and nationality) and answered the question “*on average, how many hours do you spend playing video games a week*?”. The participants then completed the CEAS, DASS-21, BACQ, and both online and offline versions of the MSPSS. The order in which these questionnaires were administered was counterbalanced to mitigate carry-over effects. Upon completion, participants were presented with a debrief sheet. Altogether, responding to the questionnaires lasted approximately 8–10 min.

## 4 Results

As shown in Table 1, the mean video game engagement score for the sample (*M* = 62.03, *SD* = 10.07) was higher than the mean addiction score (*M* = 37.55, *SD* = 14.00). A paired t-test found this difference to be significant, *t*(134) = 17.65, *p* < .01, more participants endorse criteria for highly engaged rather than addicted gaming behaviour. Whilst there were no gender differences for video game engagement, males had significantly higher video game addiction scores (*M* = 39.71, *SD* = 13.30) than females (*M* = 33.55, *SD* = 14.45), *t*(132) = 2.44, *p* = .02.

**Table 1:** Table showing descriptive statistics for all measures.

Scale	M	SD	Skew	Kurt	Range
CEAS					
Addiction	37.55	14.00	.56	-.25	13.00–75.00
Engagement	62.03	10.07	-.90	.78	29.00–78.00
DASS-21					
Depression	27.87	11.11	.98	.11	14.00–56.00
Anxiety	21.44	7.17	2.08	6.21	14.00–56.00
Stress	25.94	8.50	.85	.66	14.00–56.00
BACQ					
Approach	20.14	3.98	-.61	-.12	10.00–27.00
Diversion	9.61	2.50	-.17	-.13	3.00–15.00
Withdrawal/resignation	9.17	2.78	-.04	-.58	3.00–15.00
MSPSS					
Offline v.	4.95	1.36	-.76	.18	1.08–7.00
Online v.	4.06	1.46	-.04	-.88	1.00–6.83

As shown in Table 2, video game addiction was significantly positively correlated with depression, anxiety, and stress. In comparison, video game engagement was not correlated with any of those symptoms. Addiction was also significantly correlated with lower approach and higher withdrawal/resignation and diversion coping, whilst engagement was not significantly correlated with any of the coping measures. With regards to PSS, addiction was significantly negatively correlated with offline support, whilst engagement was significantly and positively correlated with both offline and online support. Bivariate correlations also show that addition and engagement are both significantly and positively associated with time spent playing video games weekly (see Table 3).

**Table 2:** Correlation matrix for all measures.

Scales	1	2	3	4	5	6	7	8	9	10	11	12
**CEAS**												
Addiction	—	.14	.37∗∗	.27∗∗	.35∗∗	-.29∗∗	.40∗∗	.32∗∗	-.23∗∗	-.04	.00	.35∗∗
Engagement		—	-.08	.05	.03	.01	-.11	.08	.22∗	.25∗∗	-.03	.23∗∗
**DASS-21**			—									
Depression				.53∗∗	.58∗∗	-.48∗∗	.66∗∗	.28∗∗	-.48∗∗	-.04	.08	.40∗∗
Anxiety				—	.62∗∗	-.16	.46∗∗	.21∗	-.11	.04	.09	.23∗∗
Stress					—	-.22∗	.45∗∗	.33∗∗	-.17∗	-.07	.18∗	.25∗∗
**BACQ**						—						
Approach							-.54∗∗	-.17	.56∗∗	.26∗∗	-.02	-.23∗∗
Withdrawal/resignation							—	.30∗∗	-.39∗∗	-.04	-.03	.26∗∗
Diversion								—	-.19∗	-.04	-.02	.14
**MSPSS**												
Offline									—	.16	-.01	.01
Online										—	- .21∗	.11
**Demographics**											—	
Age												.03
Weekly playing time (hours)												—

*Note*. ∗∗ = p < .01; ∗ = p < .05.

**Table 3:** Summary of constructs and results of multiple mediation analysis for each hypothesis test.

Hypothesis	Constructs	Results
H_1_	Video game addiction and engagement, DAS	• There is a total effect (positive correlation) of video game addiction and DAS. • There is no total effect of video game engagement on DAS.
H_2a_	Video game addiction DAS, avoidance and approach coping	• Coping is a full mediator of the relationship between video game addiction and DAS. • Video game addiction had an indirect effect on DAS through high withdrawal/resignation coping. • Video game addiction had an indirect effect on depression through low approach coping.
H_2b_	Video game engagement DAS, avoidance and approach coping	• Video game engagement had an indirect effect DAS through high withdrawal/resignation coping. • Video game engagement had an indirect effect on depression through low approach coping.
H_3a_	Video game addiction DAS, offline and online PSS	• Video game addiction has an indirect effect on depression through low offline PSS.
H_3b_	Video game engagement DAS, offline and online PSS	• Video game engagement has an indirect effect on depression and stress through low offline PSS.

A multiple mediation analysis was used to investigate whether the relationships of video game addiction and engagement with symptoms of depression, stress, and anxiety are mediated by coping and PSS. Using the PROCESS macro (Hayes, 2013) on SPSS 25.0, a bootstrap (5000) resample procedure was used to calculate the direct and indirect effects of engagement and addiction (independent factors) on depression, anxiety, and stress (dependent factors; see Appendix B). All statistical analyses were conducted using SPSS 25.0.

### 4.1 Validating the addiction and engagement distinction (H_1_)

There was a significant total effect of video game addiction on depression, *t*(1, 133) = 4.60, *p* < .001, R^2^ = .35, anxiety, *t*(1,133) = 3.23, *p* = .002, R^2^ = .27, and stress *t*(1,133) = 4.30, *p* < .001, R^2^ = .37 (see Figures 2.1, 3.1 and 4.1). In contrast, there were no significant total effects of video game engagement on depression, anxiety or stress (path *c; p* > .05).

**Figure 2:**
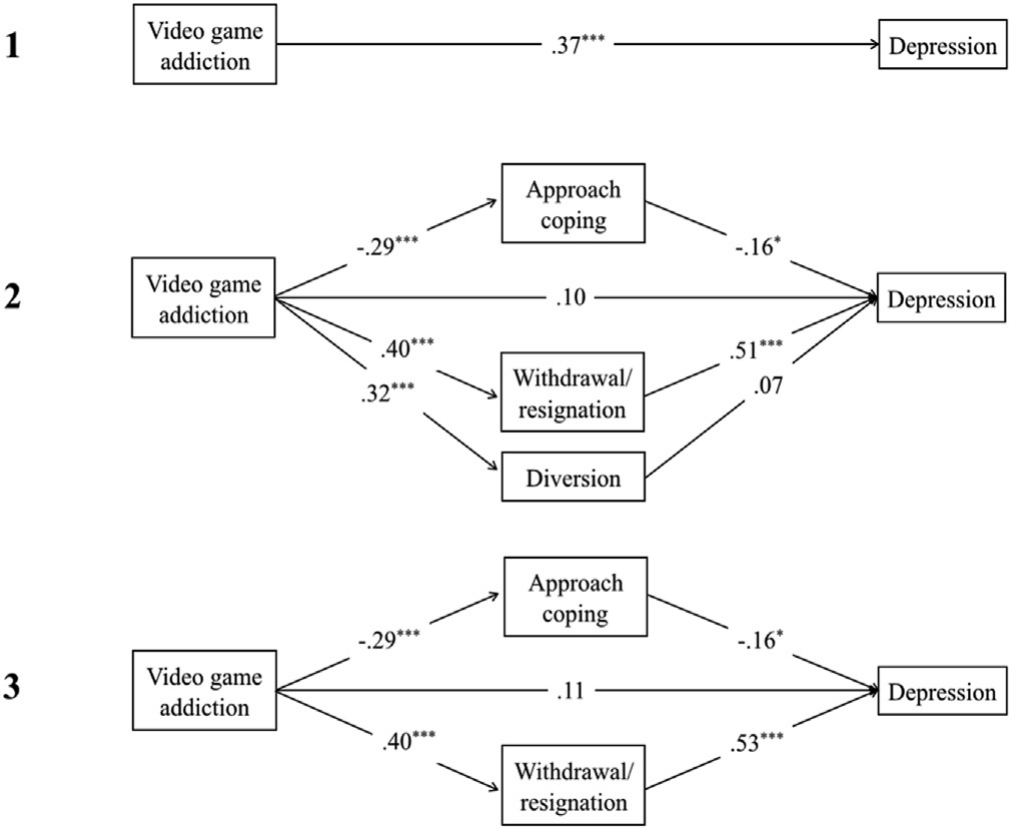
Showing standardised *β* coefficients for total, direct and indirect effects of video game addiction on depression, via coping. Figure 2.1 shows the total effect of addiction on depression. Figure 2.2 shows the multiple mediation analysis model of video game addiction and depression, mediated by approach, withdrawal/resignation, and diversion coping. Figure 2.3 shows the indirect effects of video game addiction and withdrawal/resignation on depression. *Note*. ∗∗∗ = *p* < .001; ∗∗ = *p* < .01; ∗ = *p* < .05.

**Figure 3:**
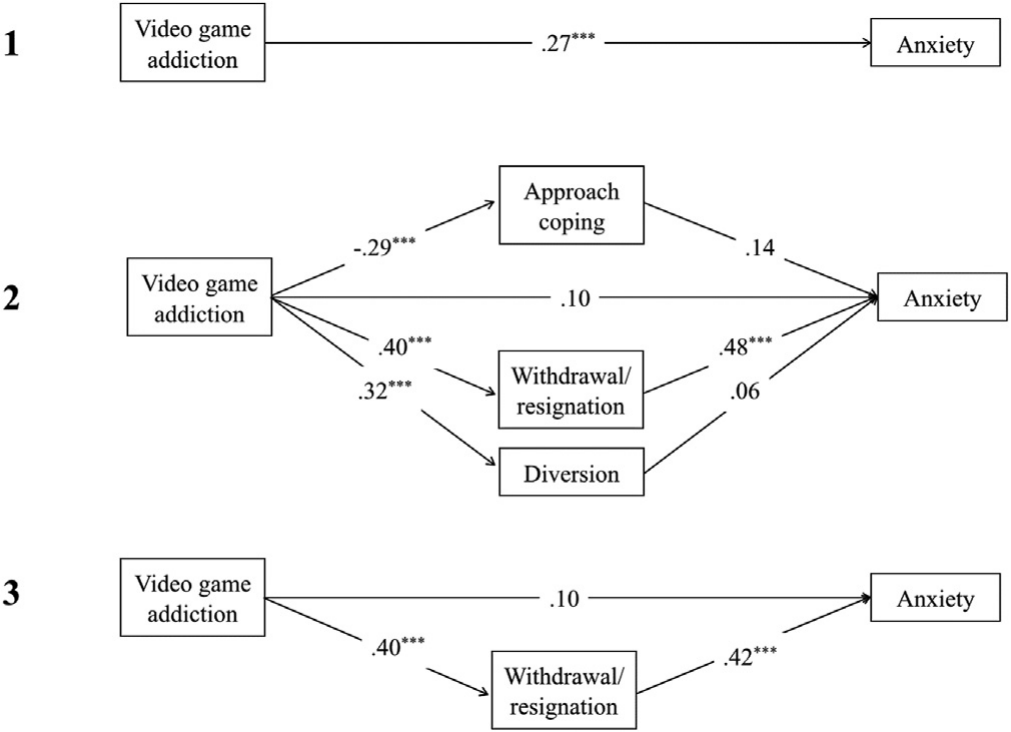
Showing standardised *β* coefficients for total, direct and indirect effects of video game addiction on anxiety, via coping. Figure 3.1 shows the total effect of addiction on anxiety. Figure 3.2 shows the multiple mediation analysis model of video game addiction and anxiety, mediated by approach, withdrawal/resignation, and diversion coping. Figure 3.3 shows the indirect effects of video game addiction and withdrawal/resignation on anxiety. *Note*. ∗∗∗ = *p* < .001; ∗∗ = *p* < .01; ∗ = *p* < .05.

**Figure 4:**
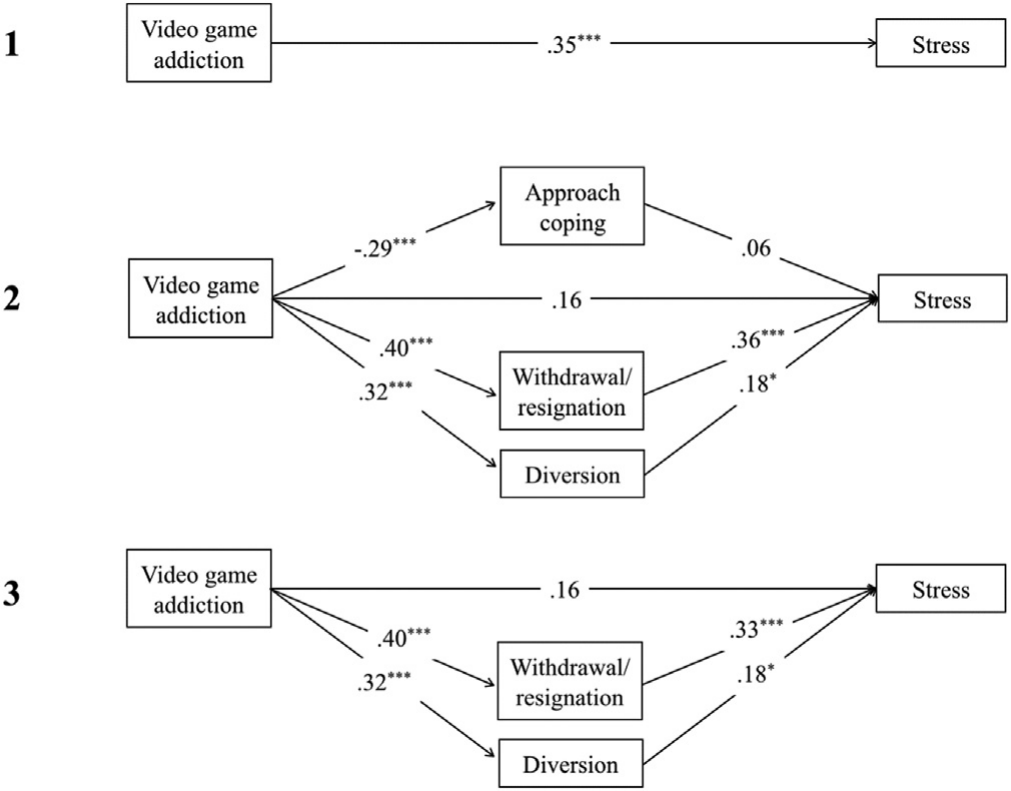
Showing standardised *β* coefficients for total, direct and indirect effects of video game addiction on stress, via coping. Figure 4.1 shows the total effect of addiction on stress. Figure 4.2 shows the multiple mediation analysis model of video game addiction and stress, mediated by approach, withdrawal/resignation, and diversion coping. Figure 4.3 shows the indirect effects of video game addiction on stress through withdrawal/resignation and diversion coping. *Note*. ∗∗∗ = *p* < .001; ∗∗ = *p* < .01; ∗ = *p* < .05.

### 4.2 The mediating role of coping (H_2a_ and H_2b_)

Higher levels of video game addiction significantly predicted lower use of approach coping, and higher use of both withdrawal/resignation and diversion coping, leading to higher depression, anxiety, and stress. When coping mediators of approach, withdrawal/resignation, and diversion were adjusted for, the direct effect of video game addiction on depression, anxiety, and stress was non-significant, (path *c'*; *p* > .05) demonstrating full mediation (see Figures 2.2, 3.2 and 4.2). Overall models were significant for depression, *F*(4,130) = 28.54, *p* < .001, R^2^ = .47, anxiety, *F*(4,130) = 10.21, *p* < .001, *R*^*2*^ = .24, and stress, *F*(4,130) = 11.89, *p* < .001, *R*^*2*^ = .27.

For depression and anxiety, the indirect effects of diversion coping were non-significant (BCa CI incorporated zero). When diversion was removed from the overall model for depression (see Figure 2.3), the significant indirect effect of addiction on depression through withdrawal/resignation coping increased, and the direct effect remained insignificant (path *c'*; *p* > .05). The same was found when diversion and approach coping were both removed from the overall model for anxiety. Both of these models were significant (depression: *F*(2,132) = 53.22, *p* < .01, R^2^ = .45; anxiety: *F*(2,132) = 18.88, *p* < .01, R^2^ = .22).

On its own, approach coping only partially mediated the relationship between addiction and depression (*β* = -.40, *p* < .01; path *c*; *p* < .01), and had no mediating effect on anxiety (see Figure 3.3). For stress, there was a significant indirect effect of addiction through diversion coping (BCa CI did not include zero; see Figure 4.2). After removing approach coping from the overall model (see Figure 4.3), the indirect effects of addiction on stress through diversion and withdrawal/resignation remained significant. This model was also significant, *F*(3,131) = 15.79, *p* < .01, R^2^ = .27.

Only withdrawal/resignation significantly mediated the relationship between video game engagement and all three symptoms of depression (*β* = .53, *p* < .01), anxiety (*β* = .53, *p* < .01) and stress (*β* = .42, *p* < .01). Approach coping also significantly mediated the link between engagement and depression (*β* = -.17, *p* = .03), in that lower approach coping predicted higher symptoms of depression for highly engaged players. For stress, diversion also emerged as a significant partial mediator (*β* = .21, *p* < .01), showing that high engagement paired with higher use of diversion coping is associated with higher stress.

### 4.3 The mediating role of social support (H_3a_ and H_3b_)

The direct effects of addiction on depression, anxiety, and stress all remained significant (path *c'*; *p* < .01) after accounting for offline and online PSS, suggesting there was no mediation effect of PSS (see Figures 5.2, 6.2 and 7.2). However, the direct effect of addiction on depression was reduced and the indirect effect of offline PSS was significant (path *b*; *p* < .01; see Figure 5.2).

**Figure 5:**
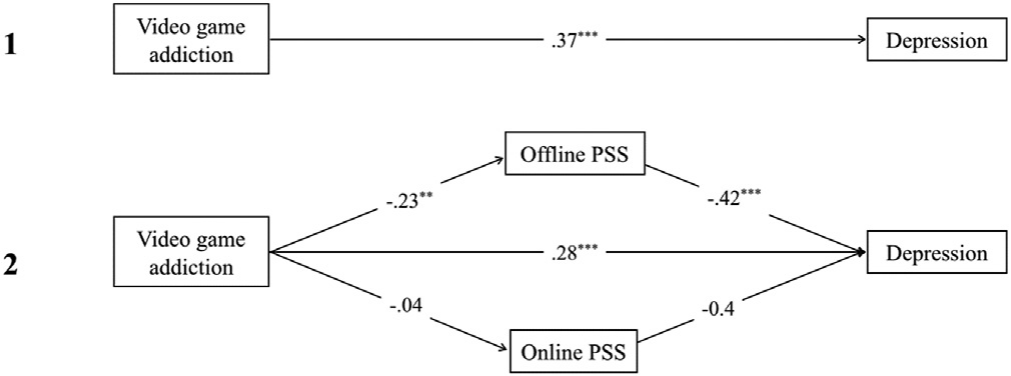
Showing standardised *β* coefficients for total, direct and indirect effects of video game addiction on depression, via PSS. Figure 5.1 shows the total effect of video game addiction on depression. Figure 5.2 shows the multiple mediation analysis model of video game addiction and depression, mediated by online and offline PSS. *Note*. ∗∗∗ = *p* < .001; ∗∗ = *p* < .01; ∗ = *p* < .05.

**Figure 6:**
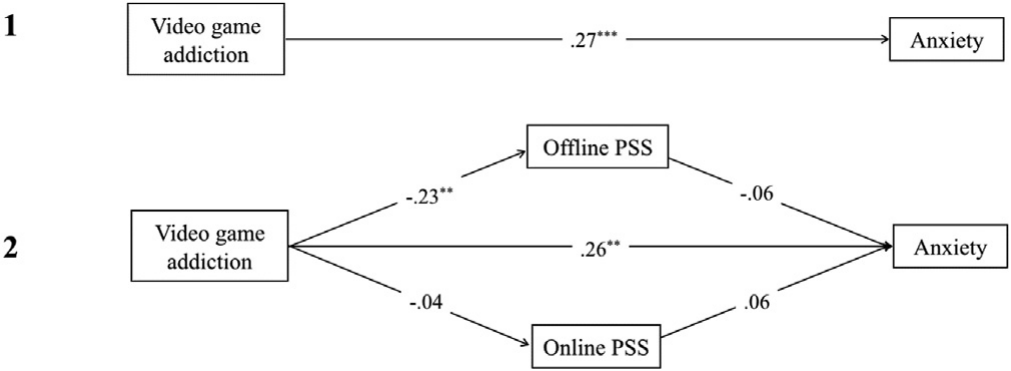
Showing standardised *β* coefficients for total, direct and indirect effects of video game addiction on anxiety, via PSS. Figure 6.1 shows the total effect of video game addiction on anxiety. Figure 6.2 shows the multiple mediation analysis model of video game addiction and anxiety, mediated by online and offline PSS. *Note*. ∗∗∗ = *p* < .001; ∗∗ = *p* < .01; ∗ = *p* < .05.

**Figure 7:**
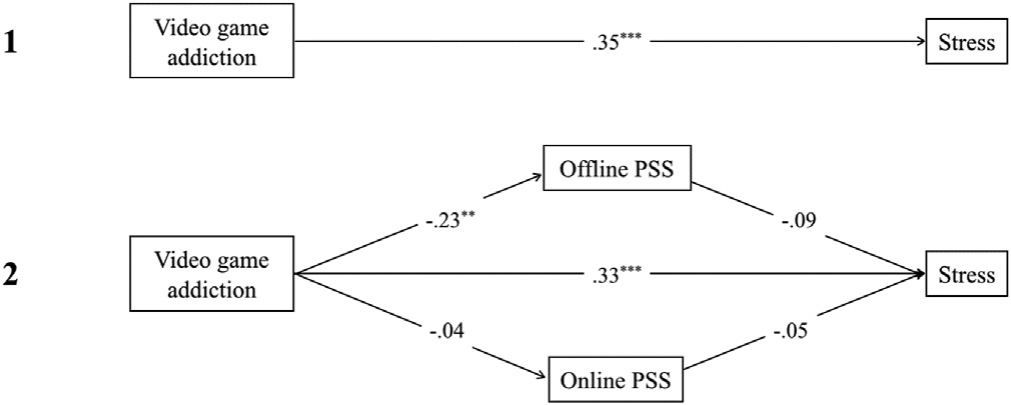
Showing standardised *β* coefficients for total, direct and indirect effects of video game addiction on stress, via PSS. Figure 7.1 shows the total effect of video game addiction on stress. Figure 7.2 shows the multiple mediation analysis model of video game addiction and stress, mediated by online and offline PSS. *Note*. ∗∗∗ = *p* < .001; ∗∗ = *p* < .01; ∗ = *p* < .05.

Concerning video game engagement, mediation models for anxiety and stress were insignificant, showing no mediation effect. However, for the model including stress, there was a significant indirect effect of offline PSS (*β =* .18, *p* = .05; BCa CI did not include zero). For depression, the overall model was significant, *F*(3,131) = 13.27, *p* < .001, R^2^ = .23. This indicated that video game engagement has a significant, positive relationship with offline PSS (path *a*; *β =* .22, *p* = .01) and that offline PSS has a significant negative relationship with depression (path *b*; *β =* -.49, *p* < .001).

## 5 Discussion

The first purpose of this study was to validate the distinction between addiction and benign engagement in video games, in the context of DAS. Supporting H_1_, analyses of total effects showed that video game addiction significantly predicted DAS, with depression showing the largest effect followed by stress and anxiety. In contrast, engagement was not related to any of those symptoms. This supports the widely reported association between video game addiction and reduced mental health (Mentzoni et al., 2011; Gentile et al., 2011; Ferguson et al., 2011), and is aligned with the proposition that computer addiction and engagement are separate constructs (Charlton, 2002; Charlton and Danforth, 2007). More importantly, the present findings show these constructs *can* be differentiated based on the occurrence of negative psychological health outcomes, agreeing with the conclusions of Loton et al. (2016) and Krossbakken et al. (2018). However, this is not completely in agreement with findings from Loton et al. (2016), who reported a significant, positive relationship between video game engagement and anxiety. Whilst researchers concluded their findings only showed partial support for Charlton and Danforth's distinction between addiction and engagement, our findings offer full support by showing (i) a positive correlation between video game addiction and all three facets of DAS, and (ii) no correlation between video game engagement and any facet of DAS. Despite being psychometrically separate conditions, we noted high inter-correlations between depression, anxiety, and stress. However, this remains consistent with their high rate of co-occurrence (Lovibond and Lovibond, 1995).

The results presented here indicate the co-occurrence of negative psychological consequences, particularly DAS symptomatology, provides a useful axis of differentiation for concepts of addiction and engagement. This is important given analyses showed addiction and engagement are both positively correlated with weekly time spent playing, adding credence to Griffiths (2010) proposition that these concepts cannot accurately not be distinguished based on time spent playing.

A second aim was to provide support for coping as a mediator in the relationship between video game addiction and mental health. Correlational analyses showed that video game addiction was significantly associated with lower approach and higher avoidance coping. Symptoms of depression and stress were also significantly associated with low approach coping, whilst DAS significantly correlated with higher withdrawal/resignation coping, followed by diversion coping. Coping emerged as a full mediator of the relationship between video game addiction and DAS. In particular, withdrawal/resignation showed the biggest mediating effect for all three outcomes of reduced mental health. In the case of depression, low approach coping additionally was found to be a significant partial mediator, and diversion was also found to be a significant partial mediator with regards to stress. These results partially support H_2a_, demonstrating that addiction has an indirect effect on DAS through high avoidance coping, however low approach coping was only significant for depression.

Video game engagement was not significantly correlated with any dimension of coping. However, there was a significant indirect effect of withdrawal/resignation on DAS. Video game engagement did not affect DAS except through high avoidance coping in the form of withdrawal/resignation, and via low approach coping for depression only. This demonstrates partial support for H2b, as low approach did not significantly mediate the paths from engagement to anxiety or stress. This suggests the use of withdrawal/resignation coping strategies is maladaptive even for individuals who score highly on measures of video game engagement.

This is aligned with previous research showing that high avoidance coping is maladaptive in the context of addiction generally (Jauregui et al., 2017; Lee-Winn et al., 2018), Internet addiction (Brand et al., 2014; McNicol and Thorsteinsson, 2017; Cheng et al., 2015; Young, 2007), and video game addiction (Plante et al., 2019). This also follows from research associating high avoidance coping with higher psychological distress and negative emotionality (Loton et al., 2016). This is because individuals choose to postpone problem-solving, only temporarily relieving stress. As in Loton et al.'s study, our results emphasise withdrawal/resignation as the most significant sub-dimension of avoidant coping. This reiterates the dissociation between withdrawal/resignation and diversion strategies within avoidance coping, with the former being a more significant mediator in the context of distress (Polman et al., 2010). Withdrawal/resignation is a form of passive avoidance, characterised by disengagement and denial. Contrary to approach coping, these include withdrawing from others, feeling like giving up, and finding it difficult to try new things (Finset et al., 2002). In the context of video game addiction, our results show this kind of avoidant behaviour contributes more significantly to poor mental health compared to escape-based diversion coping. This fits in the wider picture of gaming as a “vacation from [the] mind” (Snodgrass et al., 2014); an adaptive, cognitive diversion strategy that serves to manage negative emotions caused by offline stressors (Shi et al., 2019). However, compared to Loton et al.'s study, we also found high diversion mediated the relationship between addiction and stress, suggesting that although less significant than withdrawal/resignation, escape-based coping may remain problematic (Ballabio et al., 2017).

Lastly, approach-oriented coping may be uniquely relevant to depression symptomatology because it involves positive reinterpretation and active mobilisation of social resources. These characteristics of approach coping are in stark contrast with some core facets of depression, such as negative affect and impaired social functioning (Beck and Alford, 2009). This disparity explains why a lack of approach coping is associated with a higher endorsement of depression symptoms generally (Haskell et al., 2020), and in patients of Internet addiction (Senormanci et al., 2014).

Finally, this research explored perceived social support (PSS) as a mediator in the previously mentioned relationship. The present research identified a significant inverse correlation between video game addiction and offline PSS, and no relationship between addiction and online PSS. Whilst direct effects of addiction on mental health outcomes remained significant after the inclusion of online and offline PSS in mediation models, these were smaller than total effects. The biggest reduction was observed for addiction and depression. In this model, there was a significant negative indirect effect of offline PSS on depression. Overall, our results showed partial support for H_3a_, as the indirect effect of PSS was only significant in the context of depression and for offline PSS specifically. In contrast to addiction, engagement in video games was significantly and positively associated with both offline and online PSS. Engagement significantly leads to depression and stress only via low offline PSS, with the biggest effect being demonstrated for depression.

Our results only partially support H_3b_, as low offline PSS did not lead to symptoms of anxiety for highly engaged individuals and there were no indirect effects of online PSS. The association between lower PSS and Internet addiction (Gunuc and Dogan, 2013; Wu et al., 2016), and IGD (Wartberg et al., 2017) has been previously reported. The finding that low offline PSS specifically predicted higher levels of depression for individuals with both high video game addiction and engagement also fits with the literature on clinical depression (Zimet et al., 1988). As hypothesised by our mediation model, a causal explanation for these findings may be that all excessive gamers experience reduced face-to-face (offline) social contact, and thus support. This is also the basis of displacement theory. Given that PSS acts as a buffer for the psychological impact of stressors, lowered offline support increases vulnerability to depression. Whilst there are mixed findings regarding the benefits of online social support for mental health outcomes, our results found no indirect effects of online PSS on depression, anxiety, or stress. A possible explanation is that this study did not distinguish between on/offline video gamers. Whilst massively multiplayer online role-playing games (MMORPGs) are characterised by an inherent social aspect, offline gaming does not provide the same opportunities to receive online support from others, for example the communication component (Kraut and Burke, 2015). Nevertheless, this result corroborates findings from Tham et al. (2020) who showed that unlike offline social support, in-game support is not associated with reduced depression and anxiety for problematic gamers. Finally, high engagement was positively correlated with online PSS, which substantiates the idea that engaged players use gaming to make new social connections and connect with offline friends (Domahidi et al., 2014).

### 5.1 Theoretical and clinical implications

Validating the distinction between healthy and problematic engagement in video gameplay is a crucial step in informing the development of meaningful clinical criteria for IGD. It is also essential to accurately identify problematic gamers and avoid the conflation of healthy passion with pathology (Deleuze et al., 2018). This puts into question the utility of a polythetic classification system in the DSM-5 for IGD, in which a diagnosis is made upon endorsement of a certain number of criteria. If these criteria include both addiction and engagement factors, such a diagnostic approach may be unhelpful. Whilst clinical frameworks of addiction (e.g. Brown, 1991) have aided the conceptualisation of disordered gaming, our findings highlight that the reuse of peripheral criteria for substance use addiction (i.e. euphoria, cognitive salience, and tolerance) to define IGD may be inappropriate to uniquely diagnose pathological gaming. Our findings also suggest the Computer Engagement/Addiction Scale (CEAS; Charlton and Danforth, 2007) may be a more appropriate tool for measuring game addiction as it highlights an important difference between engagement and addiction, therefore separating excessive from pathological gamers. Other scales for example the Gaming Addiction Scale (GAS; Lemmens et al., 2009) derived from gambling research and the nine-item short form scale to assess Internet Gaming Disorder (IGD-SF9; Pontes and Griffiths, 2015) derived from the DSM-5 fail to capture this difference. Overall, following Bean et al.'s (2017) call for foundational research of problematic gaming in the context of addiction, the present findings suggest generalised addiction criteria do not necessarily translate to media consumption, by tapping into features of high engagement. By situating video game addiction within mental health, this study also emphasises the importance of considering functional impairment and distress as a key criterion distinguishing video game addiction and engagement (Billieux et al., 2017). Regarding future research, the validation of this distinction may also help warn against the overestimation of prevalence rates in population studies. Consideration of the current findings in future revisions of IGD diagnostic criteria may also foster the inclusion of clinical samples in experimental studies.

The findings presented here emphasise the importance of addressing coping in psychological interventions for video game addiction. Seeking an understanding of coping styles for individuals suffering from gaming addiction is important given that inclinations towards approach or avoidant coping strategies show cross-situational consistency (Roth and Cohen, 1986). Therefore, coping may be implicated in the long-term maintenance of video game addiction. This points towards Cognitive Behavioural Therapy (CBT), which targets poor coping responses, as a promising avenue for the treatment of gaming addiction (Griffiths and Meredith, 2009). Our findings additionally suggest the therapeutic promotion of approach coping may be less effective than encouraging the reduction of avoidance coping. This may be an aspect to consider in CBT for gaming addiction. Measures of withdrawal/resignation coping may also be useful to include in screening instruments, to identify highly engaged players at-risk of developing addiction and comorbid mental health disorders. Highly engaged players are more likely to develop symptoms of depression and stress if they perceive lower offline social support, providing another useful axis for clinicians to identify at-risk gamers early.

### 5.2 Limitations and future studies

An important limitation of this study's design is that it is cross-sectional. This is worth noting given that multiple mediation analyses, as employed here, assume a causal relationship between predictor and outcome variables. Although some longitudinal findings have supported the directionality of the relationships hypothesised in this study (i.e. Gentile et al., 2011), more research is needed to understand whether video game addiction is a cause of reduced mental health, or whether individuals with pre-existing psychological comorbidities are more likely to become addicted to gaming, or both (Liu et al., 2018). Similarly, whilst our results could be interpreted to underscore the displacement hypothesis (Kraut et al., 1998), it may be that individuals with low offline support are more likely to turn to video gameplay and later become addicted. Aside from testing Charlton (2002) hypothesis of a developmental trajectory of engagement to addiction, longitudinal designs are likely to inform the role of coping in the relationship between addiction and mental health. This is because there are significant age-related differences in coping (Folkman et al., 1987). Given much research into video game addiction has focused on adolescents, it may be pertinent to explore how their coping strategies differ from that of older age groups, and whether this is related to pathological patterns of gameplay.

Future research may supplement our current understanding of video game addiction by recruiting clinical samples and conducting clinical interviews to aid method triangulation. Whilst online self-report measures are a useful way to reach populations of video gamers which may show symptoms of addiction, as well as older populations of gamers (King et al., 2011), there remain issues with respondents' memory recall accuracy and social desirability bias in the reluctance to report negative behaviours. Given denial is a prominent coping strategy amongst adolescents at risk of developing IGD (Schneider et al., 2017), measures relying uniquely on self-report may hinder accurate diagnoses. Self-reports of mental health issues may also present a barrier for men in particular, who show significantly less willingness to report emotional difficulties (Smith et al., 2018). This is noteworthy given the high prevalence of males who engage in video gameplay compared to females (e.g. Mentzoni et al., 2011).

Another limitation of this study is related to the adaptation of the MSPSS questionnaire to create an online version. Whilst instructions invited participants to consider the support they received via the Internet; they did not make explicit that this included support received via gaming. It is also likely that individuals do not use gaming as a means to communicate with their families; this may explain why high video game addiction was not correlated with online support. In addition, the questionnaire did not make explicit whether it was referring to real-world or in-game problems; a lacking distinction which may confound evidence for the efficacy of online social support (Kaczmarek and Drążkowski, 2014). Further work is required to evaluate the validity of this questionnaire with other populations, and in general when considering social support in video gaming to distinguish between on/offline gamers.

## 6 Conclusion

This is the first study, to our knowledge, to demonstrate complete support for Charlton and Danforth (2007) distinction between addiction and engagement in gaming in the context of comorbid symptoms of depression, anxiety, and stress. Findings indicated that coping is a full mediator of the relationship between video game addiction and symptoms of depression, anxiety, and stress, with the avoidant sub-dimension of withdrawal/resignation coping showing the largest effect. In comparison, video game engagement was not directly related to any coping strategies nor any negative mental health outcomes. The results also show evidence that low offline perceived social support partially mediated the relationship between video game addiction and depression and may also present a risk for highly engaged gamers. Comparatively, online perceived social support may be less important. Together, these findings contextualise the relationship between video game addiction/engagement in mental health, and point towards the consideration of coping strategies, and offline social support to a lesser extent, in clinical interventions for video game addiction.

## Declarations

### Author contribution statement

C. E. Moge: Conceived and designed the experiments; Performed the experiments; Analyzed and interpreted the data; Contributed reagents, materials, analysis tools or data; Wrote the paper.

D. M. Romano: Conceived and designed the experiments; Analyzed and interpreted the data; Wrote the paper.

### Funding statement

This research did not receive any specific grant from funding agencies in the public, commercial, or not-for-profit sectors.

### Competing interest statement

The authors declare no conflict of interest.

### Additional information

No additional information is available for this paper.

## Appendix A Supplementary data

Heliyon Supplementary Material - Appendices.docx Heliyon Supplementary Material - Appendices.docx

## References

[bib1] Allison S.E., Von Wahlde L., Shockley T., Gabbard G.O. (2006). The development of the self in the era of the internet and role-playing fantasy games. Am. J. Psychiatr..

[bib2] American Psychiatric Association (2013). Diagnostic and Statistical Manual of Mental Disorders (DSM-5®). https://www.academia.edu/download/38718268/CSL6820_21.pdf.

[bib3] Ballabio M., Griffiths M.D., Urbán R., Quartiroli A., Demetrovics Z., Király O. (2017). Do gaming motives mediate between psychiatric symptoms and problematic gaming? An empirical survey study. Addiction Res. Theor..

[bib86] Bean A.M., Nielsen R.K., Van Rooij A.J., Ferguson C.J. (2017). Video game addiction: The push to pathologize video games.. Professional Psychology: Research and Practice.

[bib4] Beck A.T., Alford B.A. (2009). Depression: Causes and Treatment.

[bib5] Beranuy M., Carbonell X., Griffiths M.D. (2013). A qualitative analysis of online gaming addicts in treatment. Int. J. Ment. Health Addiction.

[bib6] Billieux J., King D.L., Higuchi S., Achab S., Bowden-Jones H., Hao W., Poznyak V. (2017). Functional impairment matters in the screening and diagnosis of gaming disorder: commentary on: scholars’ open debate paper on the World Health Organization ICD-11 Gaming Disorder proposal (Aarseth et al.). J. Behav. Addict..

[bib7] Billings A.G., Moos R.H. (1981). The role of coping responses and social resources in attenuating the stress of life events. J. Behav. Med..

[bib89] Blais J.J., Craig W.M., Pepler D., Connolly J. (2008). Adolescents online: The importance of Internet activity choices to salient relationships.. ournal of youth and adolescence.

[bib8] Brand M., Laier C., Young K.S. (2014). Internet addiction: coping styles, expectancies, and treatment implications. Front. Psychol..

[bib9] Brown R.I.F., Kerr J.H., Apter M.J. (1991). Gaming, gambling and other addictive play. Adult Play: A Reversal Theory Approach.

[bib10] Brunborg G.S., Mentzoni R.A., Melkevik O.R., Torsheim T., Samdal O., Hetland J., Palleson S. (2013). Gaming addiction, gaming engagement, and psychological health complaints among Norwegian adolescents. Media Psychol..

[bib11] Brunborg G.S., Mentzoni R.A., Frøyland L.R. (2014). Is video gaming, or video game addiction, associated with depression, academic achievement, heavy episodic drinking, or conduct problems?. J. Behav. Addictions.

[bib12] Chappell D., Eatough V., Davies M.N., Griffiths M. (2006). EverQuest—it’s just a computer game right? An interpretative phenomenological analysis of online gaming addiction. Int. J. Ment. Health Addiction.

[bib13] Charlton J.P. (2002). A factor-analytic investigation of computer ‘addiction’and engagement. Br. J. Psychol..

[bib14] Charlton J.P., Birkett P.E. (1995). The development and validation of the computer Apathy and anxiety scale. J. Educ. Comput. Res..

[bib15] Charlton J.P., Danforth I.D. (2007). Distinguishing addiction and high engagement in the context of online game playing. Comput. Hum. Behav..

[bib16] Cheng C., Sun P., Mak K.K. (2015). Internet addiction and psychosocial maladjustment: avoidant coping and coping inflexibility as psychological mechanisms. Cyberpsychol. Behav. Soc. Netw..

[bib17] Coyne S.M., Stockdale L.A., Warburton W., Gentile D.A., Yang C., Merrill B.M. (2020). Pathological video game symptoms from adolescence to emerging adulthood: a 6-year longitudinal study of trajectories, predictors, and outcomes. Dev. Psychol..

[bib18] Deleuze J., Long J., Liu T.Q., Maurage P., Billieux J. (2018). Passion or addiction? Correlates of healthy versus problematic use of videogames in a sample of French-speaking regular players. Addict. Behav..

[bib19] Domahidi E., Festl R., Quandt T. (2014). To dwell among gamers: investigating the relationship between social online game use and gaming-related friendships. Comput. Hum. Behav..

[bib20] Ferguson C.J., Colwell J. (2020). Lack of consensus among scholars on the issue of video game “addiction”. Psychol. Popular Media.

[bib21] Ferguson C.J., Coulson M., Barnett J. (2011). A meta-analysis of pathological gaming prevalence and comorbidity with mental health, academic and social problems. J. Psychiatr. Res..

[bib22] Finset A., Steine S., Haugli L., Steen E., Laerum E. (2002). The brief approach/avoidance coping questionnaire: development and validation. Psychol. Health Med..

[bib23] Folkman S., Lazarus R.S., Gruen R.J., DeLongis A. (1986). Appraisal, coping, health status, and psychological symptoms. J. Pers. Soc. Psychol..

[bib24] Folkman S., Lazarus R.S., Pimley S., Novacek J. (1987). Age differences in stress and coping processes. Psychol. Aging.

[bib25] Gentile D.A., Choo H., Liau A., Sim T., Li D., Fung D., Khoo A. (2011). Pathological video game use among youths: a two-year longitudinal study. Pediatrics.

[bib26] Griffiths M.D., Meredith A. (2009). Videogame addiction and its treatment. J. Contemp. Psychother..

[bib27] Griffiths M.D. (2010). The role of context in online gaming excess and addiction: some case study evidence. Int. J. Ment. Health Addiction.

[bib28] Gunuc S., Dogan A. (2013). The relationships between Turkish adolescents’ Internet addiction, their perceived social support and family activities. Comput. Hum. Behav..

[bib29] Haskell A.M., Britton P.C., Servatius R.J. (2020). Toward an assessment of escape/avoidance coping in depression. Behav. Brain Res..

[bib30] Hayes A.F. (2013). The PROCESS Macro for SPSS and SAS.

[bib31] Herrero Olaizola J.B., Torres A.V., Vivas P., Urueña A. (2019). Smartphone addiction and social support: a three-year longitudinal study. Psychosoc. Interv..

[bib32] House J.S. (1983). Work Stress and Social Support.

[bib33] Jauregui P., Onaindia J., Estévez A. (2017). Adaptive and maladaptive coping strategies in adult pathological gamblers and their mediating role with anxious-depressive symptomatology. J. Gambl. Stud..

[bib34] Jeong E.J., Kim D.H. (2011). Social activities, self-efficacy, game attitudes, and game addiction. Cyberpsychol., Behav. Soc. Netw..

[bib87] Kaczmarek L.D., Drążkowski D. (2014). MMORPG escapism predicts decreased well-being: Examination of gaming time, game realism beliefs, and online social support for offline problems.. Cyberpsychology, Behavior, and Social Networking.

[bib35] Katz J.E., Aspden P. (1997). A nation of strangers?. Commun. ACM.

[bib36] Kim S.J., Marsch L.A., Brunette M.F., Dallery J. (2017). Harnessing Facebook for smoking reduction and cessation interventions: Facebook user engagement and social support predict smoking reduction. J. Med. Internet Res..

[bib37] King D.L., Delfabbro P.H., Zajac I.T. (2011). Preliminary validation of a new clinical tool for identifying problem video game playing. Int. J. Ment. Health Addiction.

[bib38] Kraut R., Burke M. (2015). Internet use and psychological well-being: effects of activity and audience. Commun. ACM.

[bib39] Kraut R., Patterson M., Lundmark V., Kiesler S., Mukophadhyay T., Scherlis W. (1998). Internet paradox: a social technology that reduces social involvement and psychological well-being?. Am. Psychol..

[bib40] Krossbakken E., Pallesen S., Mentzoni R.A., King D.L., Molde H., Finserås T.R., Torsheim T. (2018). A cross-lagged study of developmental trajectories of video game engagement, addiction, and mental health. Front. Psychol..

[bib41] Kuss D.J., Griffiths M.D. (2012). Internet gaming addiction: a systematic review of empirical research. Int. J. Ment. Health Addiction.

[bib43] Lee-Winn A.E., Mendelson T., Johnson R.M. (2018). Associations between coping and marijuana use in a nationally representative sample of adolescents in the United States. Addict. Behav..

[bib44] Lemmens J.S., Valkenburg P.M., Peter J. (2009). Development and validation of a game addiction scale for adolescents. Media Psychol..

[bib45] Liu L., Yao Y.W., Li C.S.R., Zhang J.T., Xia C.C., Lan J.,., Fang X.Y. (2018). The comorbidity between internet gaming disorder and depression: interrelationship and neural mechanisms. Front. Psychiatr..

[bib46] Lopez-Fernandez O., Williams A.J., Griffiths M.D., Kuss D.J. (2019). Female gaming, gaming addiction, and the role of women within gaming culture: a narrative literature review. Front. Psychiatr..

[bib47] Loton D., Borkoles E., Lubman D., Polman R. (2016). Video game addiction, engagement and symptoms of stress, depression and anxiety: the mediating role of coping. Int. J. Ment. Health Addiction.

[bib48] Lovibond P.F., Lovibond S.H. (1995). The structure of negative emotional states: comparison of the depression anxiety stress scales (DASS) with the Beck depression and anxiety inventories. Behav. Res. Ther..

[bib49] McNicol M.L., Thorsteinsson E.B. (2017). Internet addiction, psychological distress, and coping responses among adolescents and adults. Cyberpsychol. Behav. Soc. Netw..

[bib50] Mentzoni R.A., Brunborg G.S., Molde H., Myrseth H., Skouverøe K.J.M., Hetland J., Pallesen S. (2011). Problematic video game use: estimated prevalence and associations with mental and physical health. Cyberpsychol. Behav. Soc. Netw..

[bib51] Miyata K., Kobayashi T. (2008). Causal relationship between Internet use and social capital in Japan. Asian J. Soc. Psychol..

[bib52] Morozova M., Rabin R.A., George T.P. (2015). Co-morbid tobacco use disorder and depression: a re-evaluation of smoking cessation therapy in depressed smokers. Am. J. Addict..

[bib53] Muriel D., Crawford G. (2018). Video Games as Culture: Considering the Role and Importance of Video Games in Contemporary Society.

[bib54] Neupane S.P. (2016). Neuroimmune interface in the comorbidity between alcohol use disorder and major depression. Front. Immunol..

[bib55] Nunnally J., Bernstein I. (1995). Psychometric theory applied. Psychological Measurement.

[bib56] Nuyens F.M., Kuss D.J., Lopez-Fernandez O., Griffiths M.D. (2019). The empirical analysis of non-problematic video gaming and cognitive skills: a systematic review. Int. J. Ment. Health Addiction.

[bib57] Petry N.M., Weiss L. (2009). Social support is associated with gambling treatment outcomes in pathological gamblers. Am. J. Addict..

[bib59] Plante C.N., Gentile D.A., Groves C.L., Modlin A., Blanco-Herrera J. (2019). Video games as coping mechanisms in the etiology of video game addiction. Psychol. Popular Media Cult..

[bib60] Polman R., Borkoles E., Nicholls A.R. (2010). Type D personality, stress, and symptoms of burnout: the influence of avoidance coping and social support. Br. J. Health Psychol..

[bib61] Pontes H.M., Griffiths M.D. (2015). Measuring DSM-5 Internet gaming disorder: development and validation of a short psychometric scale. Comput. Hum. Behav..

[bib62] Reinhardt J.P., Boerner K., Horowitz A. (2006). Good to have but not to use: differential impact of perceived and received support on well-being. J. Soc. Pers. Relat..

[bib63] Roth S., Cohen L.J. (1986). Approach, avoidance, and coping with stress. Am. Psychol..

[bib64] Saunders J.B., Hao W., Long J., King D.L., Mann K., Fauth-Bühler M.,., Chan E. (2017). Gaming disorder: its delineation as an important condition for diagnosis, management, and prevention. J. Behav. Addictions.

[bib88] Schneider L.A., King D.L., Delfabbro P.H. (2017). Family factors in adolescent problematic Internet gaming: A systematic review.. Journal of Behavioral Addictions.

[bib65] Seah M.L., Cairns P. (2008). From immersion to addiction invideogames. People Comput. XXII Cult. Creativ. Interact..

[bib66] Senormanci O., Konkan R., Güçlü O., Senormanci G. (2014). Evaluation of coping strategies of male patients, being treated in internet addiction outpatient clinic in Turkey. J. Mood Disord..

[bib67] Seok S., DaCosta B. (2014). Distinguishing addiction from high engagement: an investigation into the social lives of adolescent and young adult massively multiplayer online game players. Game. Cult..

[bib68] Shi J., Renwick R., Turner N.E., Kirsh B. (2019). Understanding the lives of problem gamers: the meaning, purpose, and influences of video gaming. Comput. Hum. Behav..

[bib69] Sirois F.M., Kitner R. (2015). Less adaptive or more maladaptive? A meta-analytic investigation of procrastination and coping. Eur. J. Pers..

[bib70] Smith D.T., Mouzon D.M., Elliott M. (2018). Reviewing the assumptions about men’s mental health: an exploration of the gender binary. Am. J. Men's Health.

[bib71] Snodgrass J.G., Lacy M.G., Dengah H.F., Eisenhauer S., Batchelder G., Cookson R.J. (2014). A vacation from your mind: problematic online gaming is a stress response. Comput. Hum. Behav..

[bib72] Stockdale L., Coyne S.M. (2018). Video game addiction in emerging adulthood: cross-sectional evidence of pathology in video game addicts as compared to matched healthy controls. J. Affect. Disord..

[bib73] Sweeny K., Reynolds C.A., Falkenstein A., Andrews S.E., Dooley M.D. (2016). Two definitions of waiting well. Emotion.

[bib74] Tang J., Yu Y., Du Y., Ma Y., Zhang D., Wang J. (2014). Prevalence of internet addiction and its association with stressful life events and psychological symptoms among adolescent internet users. Addict. Behav..

[bib75] Taylor S.E., Stanton A.L. (2007). Coping resources, coping processes, and mental health. Annu. Rev. Clin. Psychol..

[bib76] Tham S.M., Ellithorpe M., Meshi D. (2020). Real-world social support but not in- game social support is related to reduced depression and anxiety associated with problematic gaming. Addict. Behav..

[bib77] Thompson G., McBride R.B., Hosford C.C., Halaas G. (2016). Resilience among medical students: the role of coping style and social support. Teach. Learn. Med..

[bib78] Uchino B.N. (2009). Understanding the links between social support and physical health: a life-span perspective with emphasis on the separability of perceived and received support. Perspect. Psychol. Sci..

[bib79] Vermeulen L., Van Looy J. (2016). “I play so I am?” A gender study into stereotype perception and genre choice of digital game players. J. Broadcast. Electron. Media.

[bib80] Wartberg L., Kriston L., Kramer M., Schwedler A., Lincoln T.M., Kammerl R. (2017). Internet gaming disorder in early adolescence: associations with parental and adolescent mental health. Eur. Psychiatr..

[bib81] Wartberg L., Kriston L., Zieglmeier M., Lincoln T., Kammerl R. (2019). A longitudinal study on psychosocial causes and consequences of Internet gaming disorder in adolescence. Psychol. Med..

[bib82] World Health Organisation (2018). Gaming Disorder. https://www.who.int/features/qa/gaming-disorder/en/.

[bib83] Wu X.S., Zhang Z.H., Zhao F., Wang W.J., Li Y.F., Bi L.,., Gong F.F. (2016). Prevalence of Internet addiction and its association with social support and other related factors among adolescents in China. J. Adolesc..

[bib84] Young K.S. (2007). Cognitive behavior therapy with Internet addicts: treatment outcomes and implications. Cyberpsychol. Behav..

[bib85] Zimet G.D., Dahlem N.W., Zimet S.G., Farley G.K. (1988). The multidimensional scale of perceived social support. J. Pers. Assess..

