# Supplementary material for: Contextualising video game engagement and addiction in mental health: the mediating roles of coping and social support
Source: Heliyon. 2020 Nov 16;6(11):e05340. doi: 10.1016/j.heliyon.2020.e05340 (PMC7672223; doi:10.1016/j.heliyon.2020.e05340)

**Appendix A**

Multi-dimensional Scale of Perceived Online Social Support

*Instruction: “We are interested in how you feel about the following statements. Read each statement carefully and indicate how you feel. Consider each of the following statements only in relation to online contexts (e.g. think of the support you receive from phone calls, texts and Internet use).”*

1. There is an online special person who is around when I am in need.
2. There is an online special person with whom I can share my joys and sorrows.
3. My family really tries to help me via phone calls, text messaging or the Internet.
4. I get the emotional help and support I need from my family via phone calls, text messaging or the Internet.
5. I have an online special person who is real source of comfort for me.
6. My online friends really try to help me.
7. I can count on my online friends when things go wrong.
8. I can talk about my problems with my family via phone calls, text messaging or through the Internet.
9. I have online friends with whom I can share my joys and sorrows.
10. There is an online special person in my life who cares about my feelings.
11. My family is willing to help me make decisions, via phone calls, text messaging or the Internet.
12. I can talk about my problems with my online friends.

**Appendix B**

SPSS data

Models 1-3: Addiction, DAS and coping

Models 4-6: Engagement, DAS and coping

Models 7-9: Addiction, DAS and PSS

Models 10-12: Engagement, DAS and PSS

**Model 1** (corresponding to Figure 2 in text)

Addiction

Depression

Diversion

Withdrawal

Approach


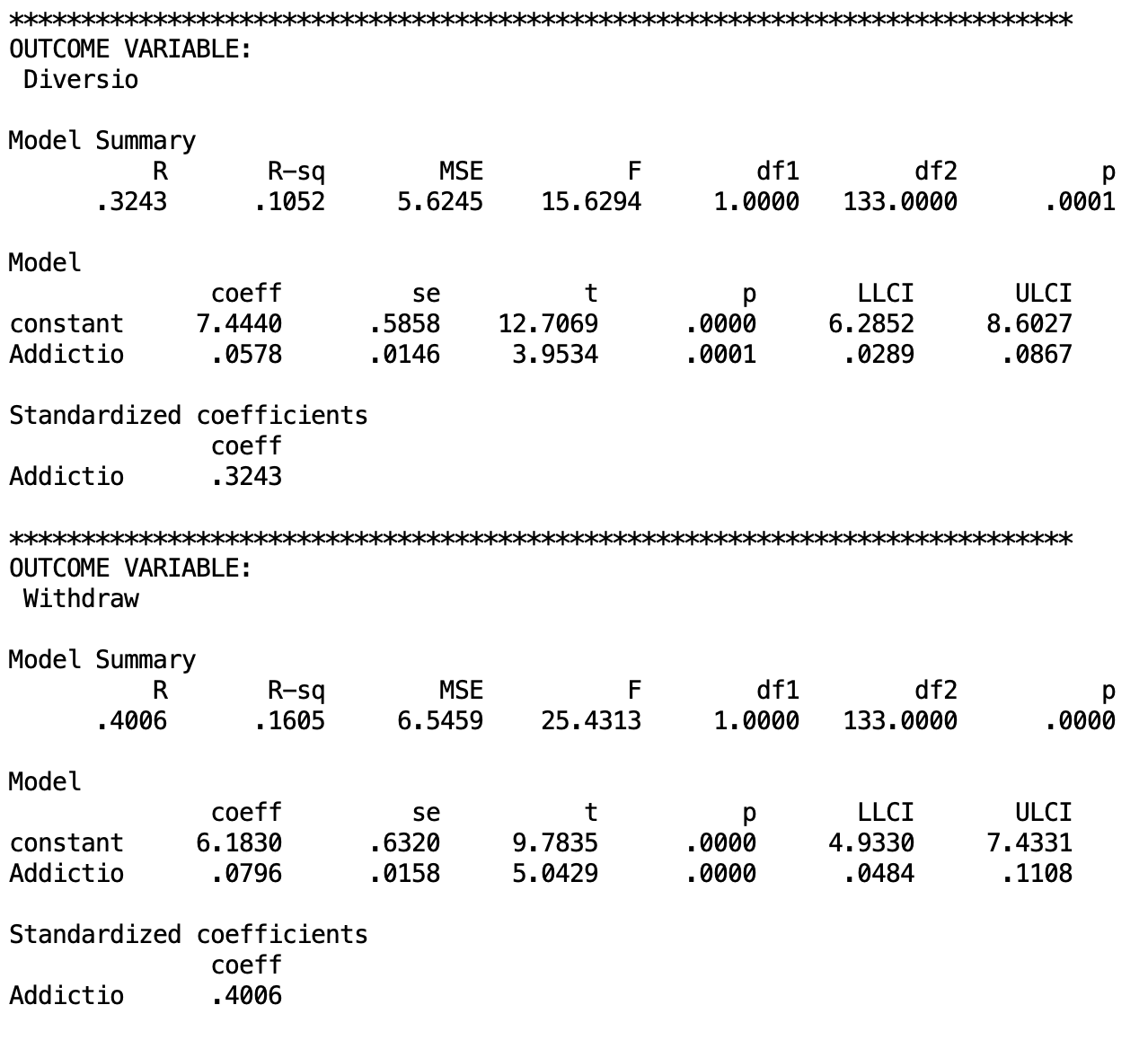


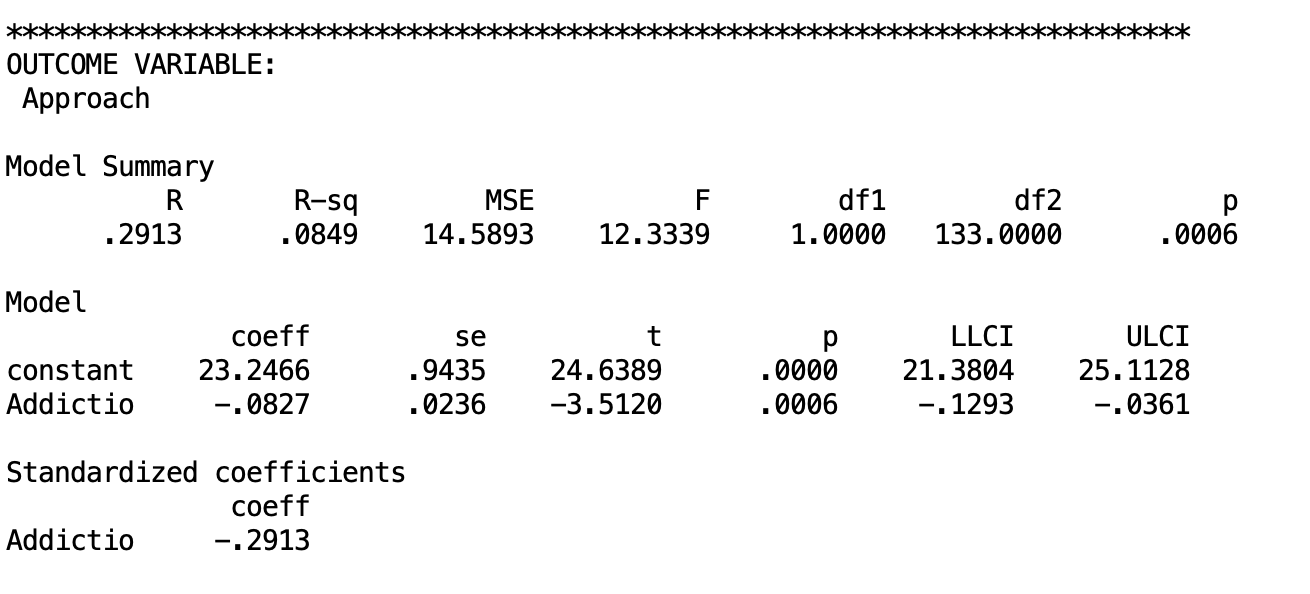


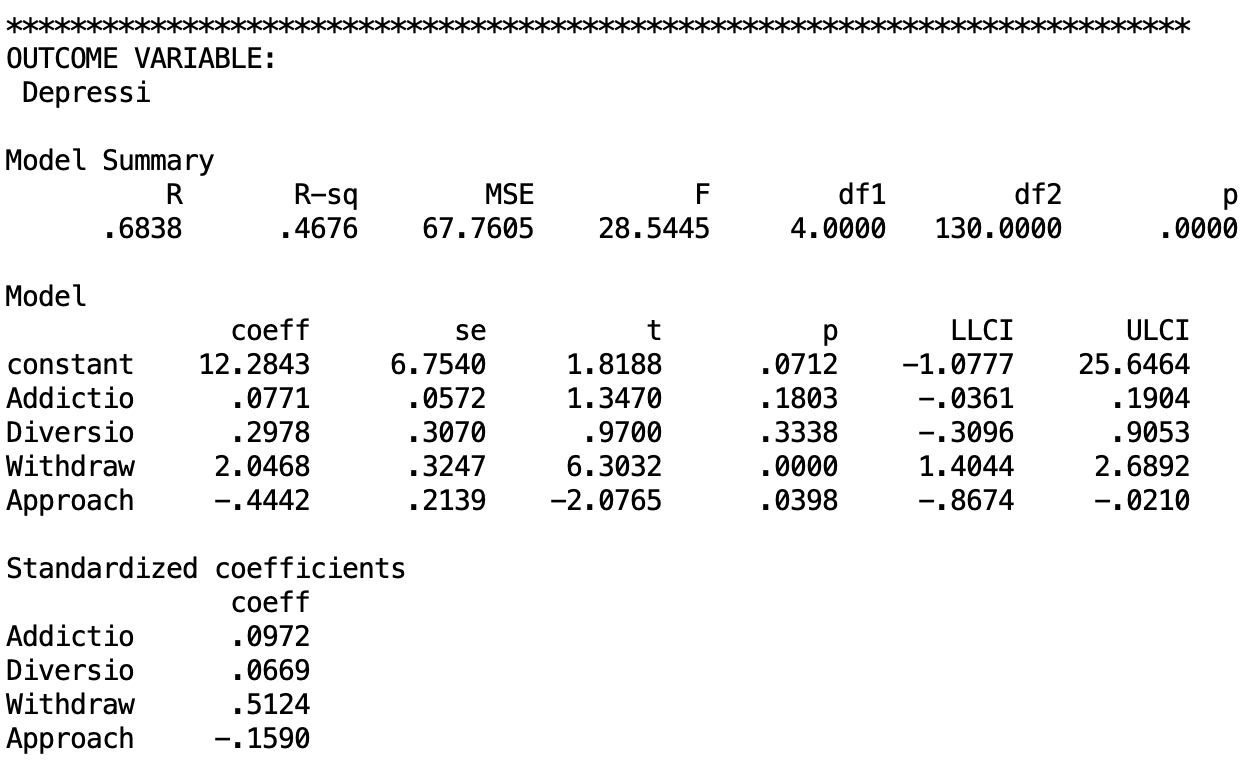


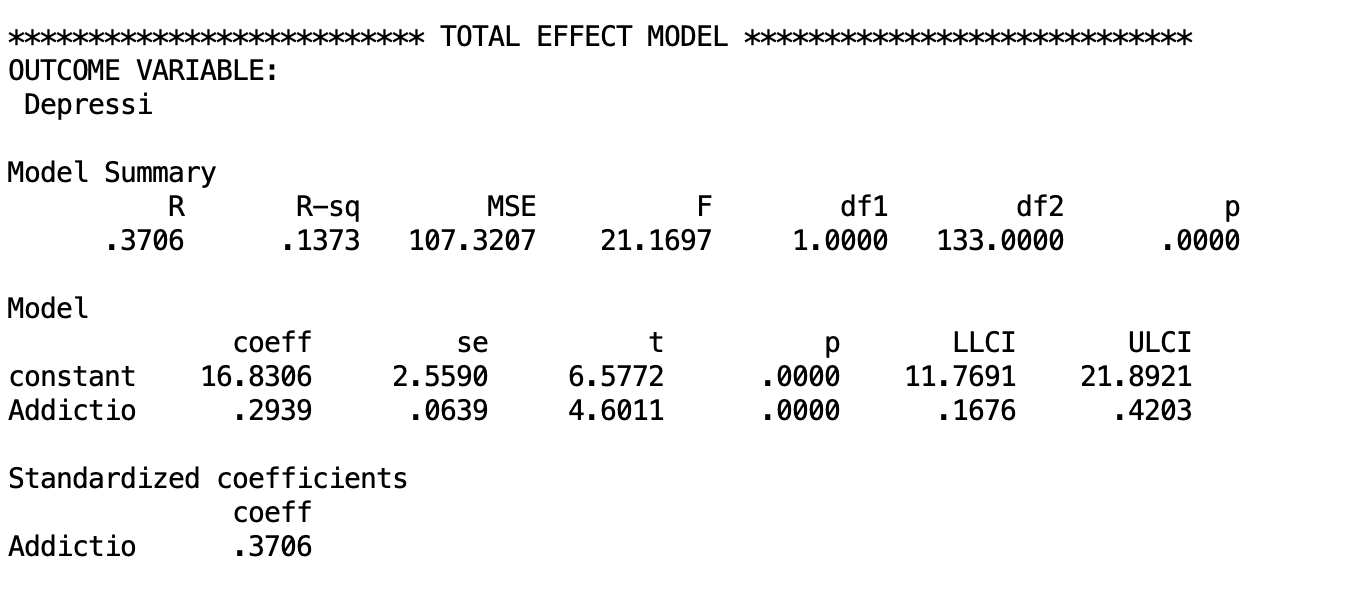


After removal of diversion coping:


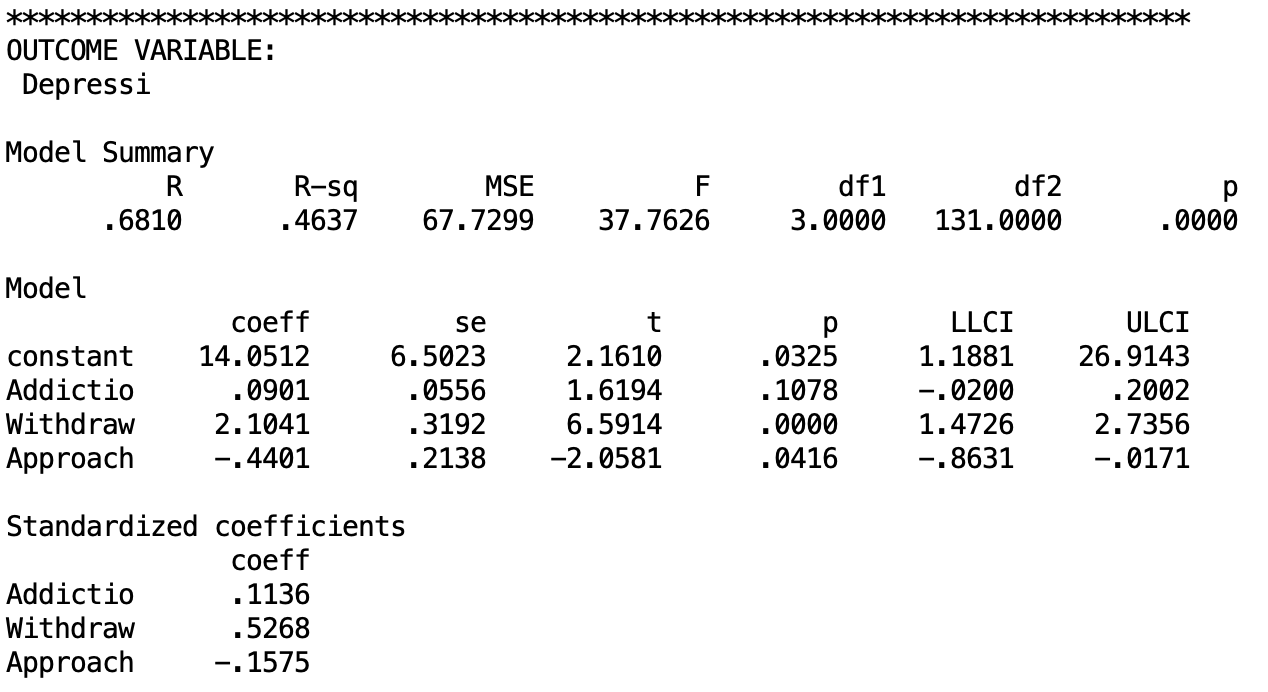


**Model 2** (corresponding to Figure 3 in text)

Addiction

Anxiety

Diversion

Withdrawal

Approach


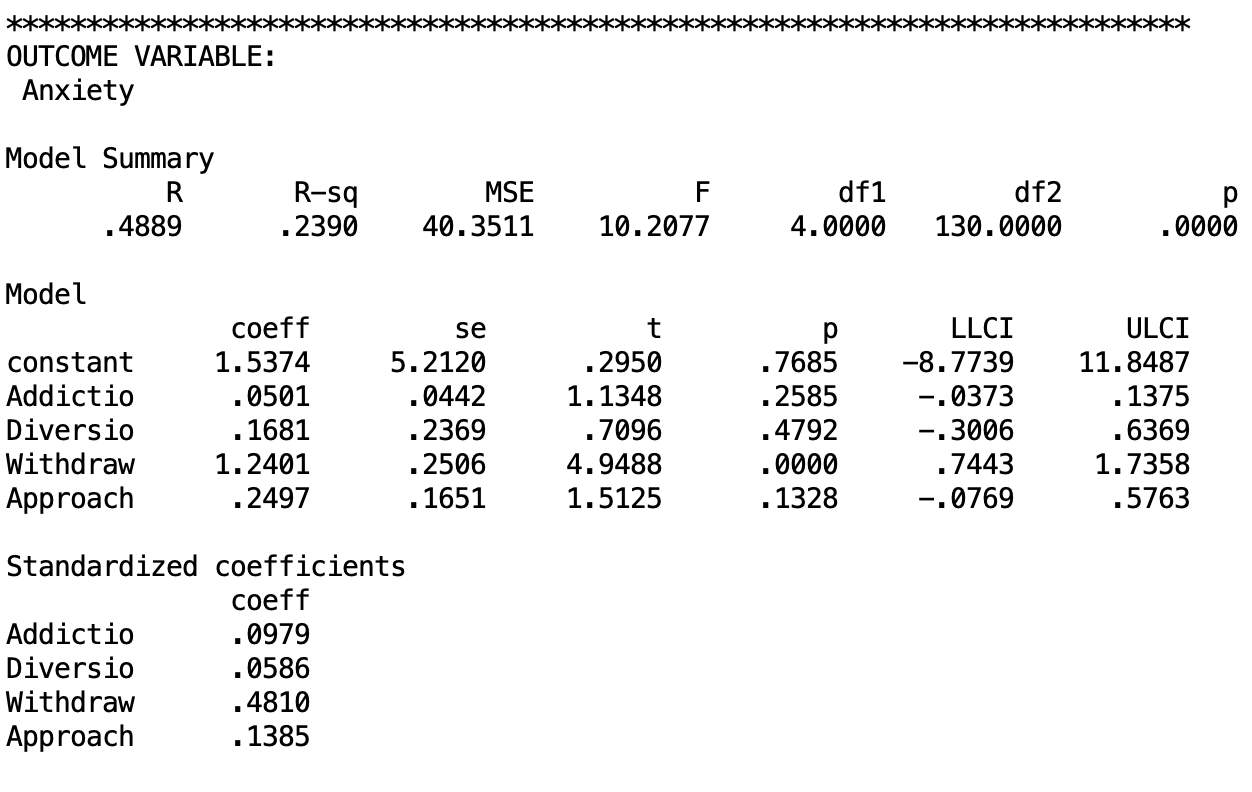


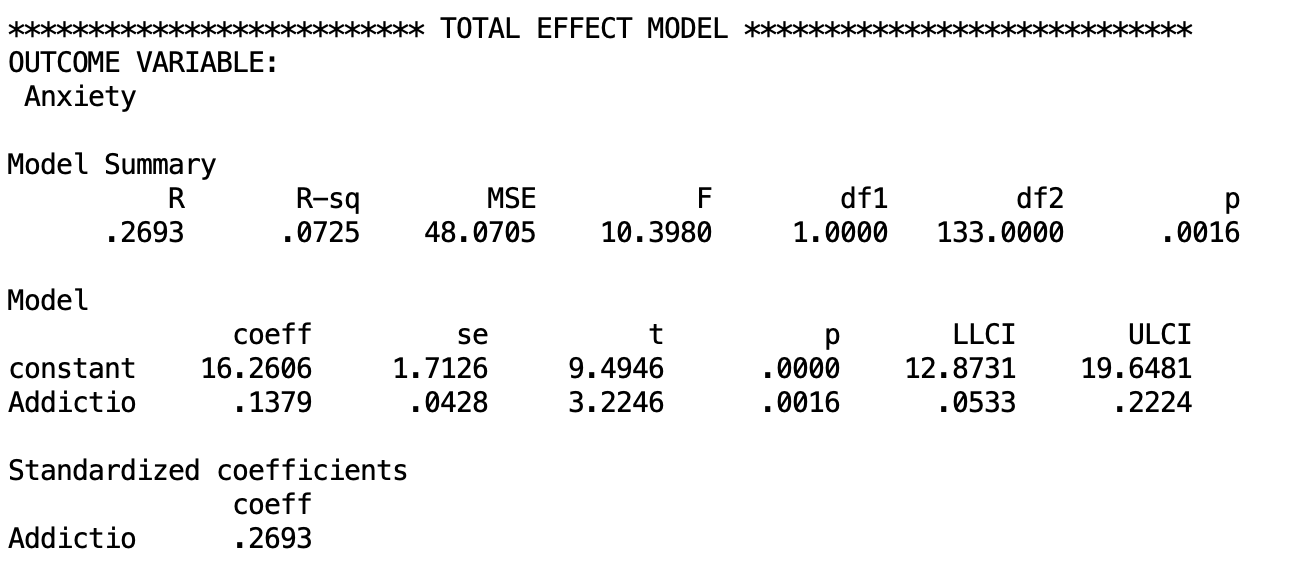


After removal of diversion and approach coping:


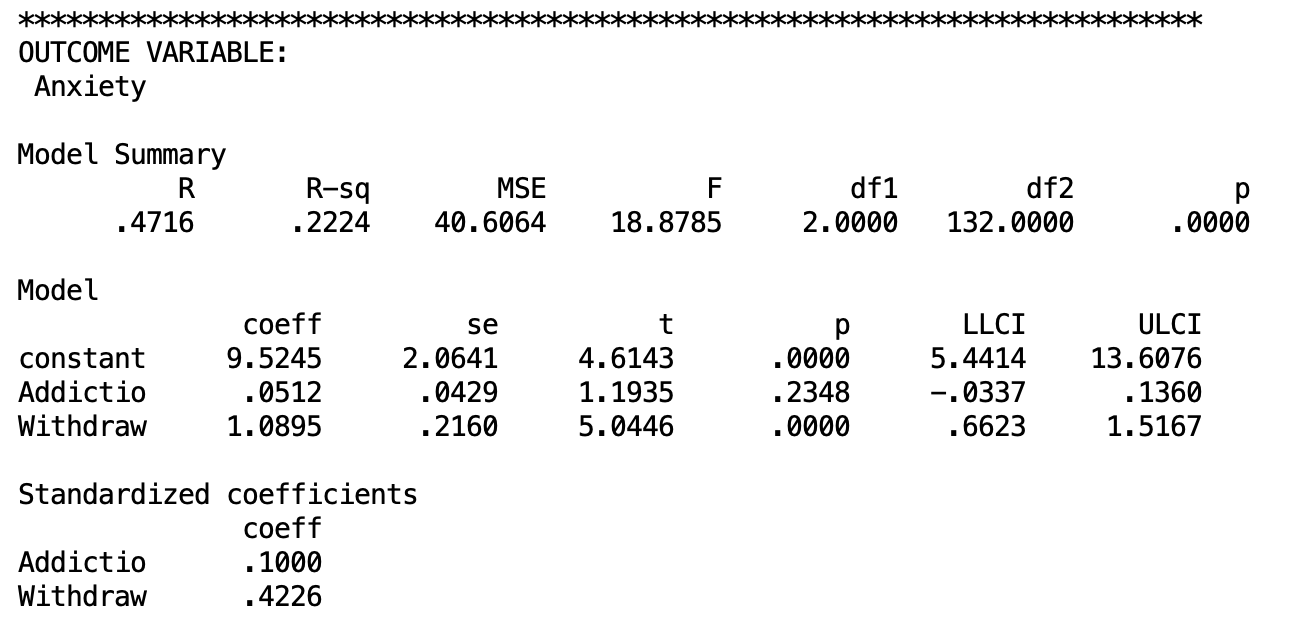


**Model 3** (corresponding to Figure 4 in text)

Addiction

Stress

Diversion

Withdrawal

Approach


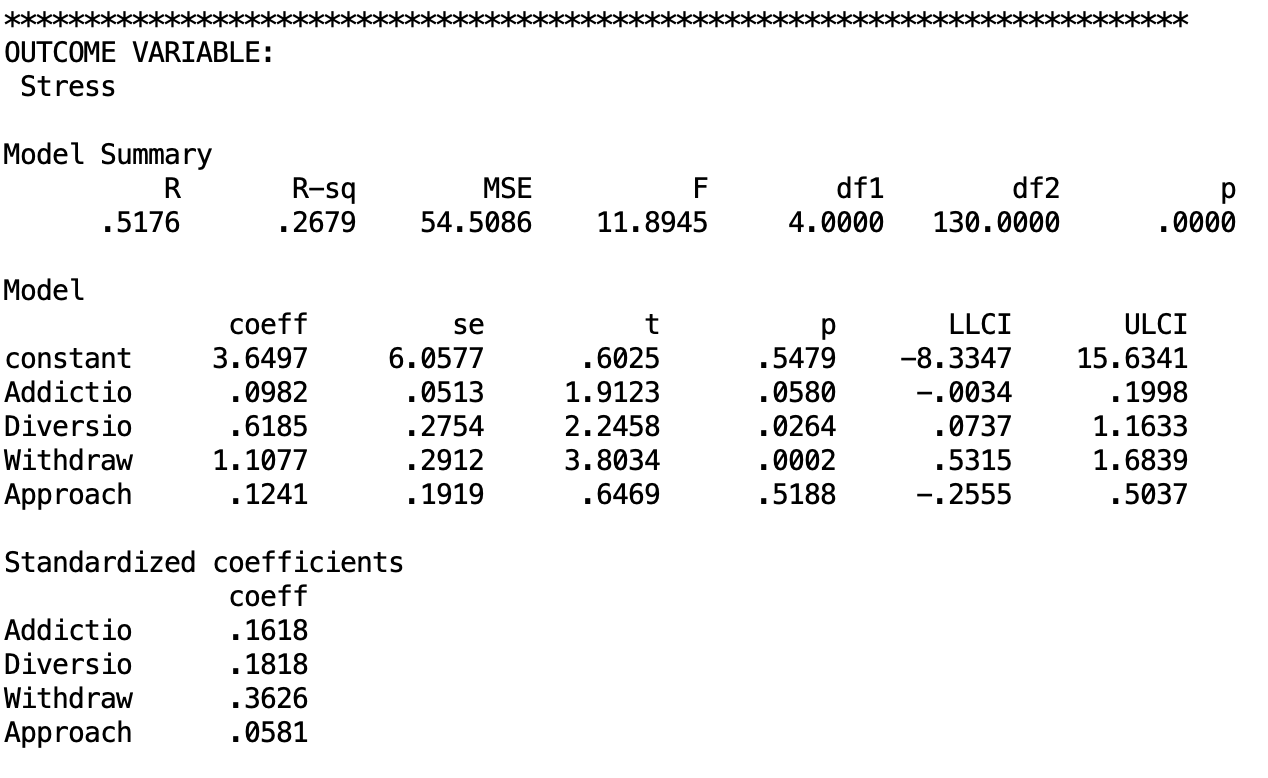


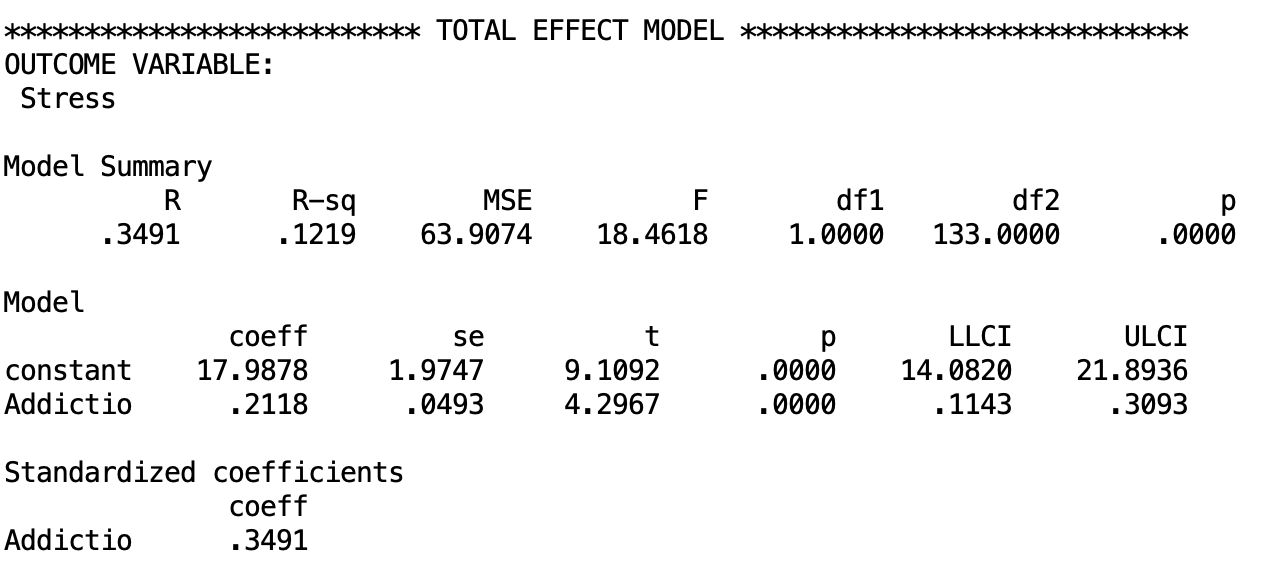


After removal of approach coping:


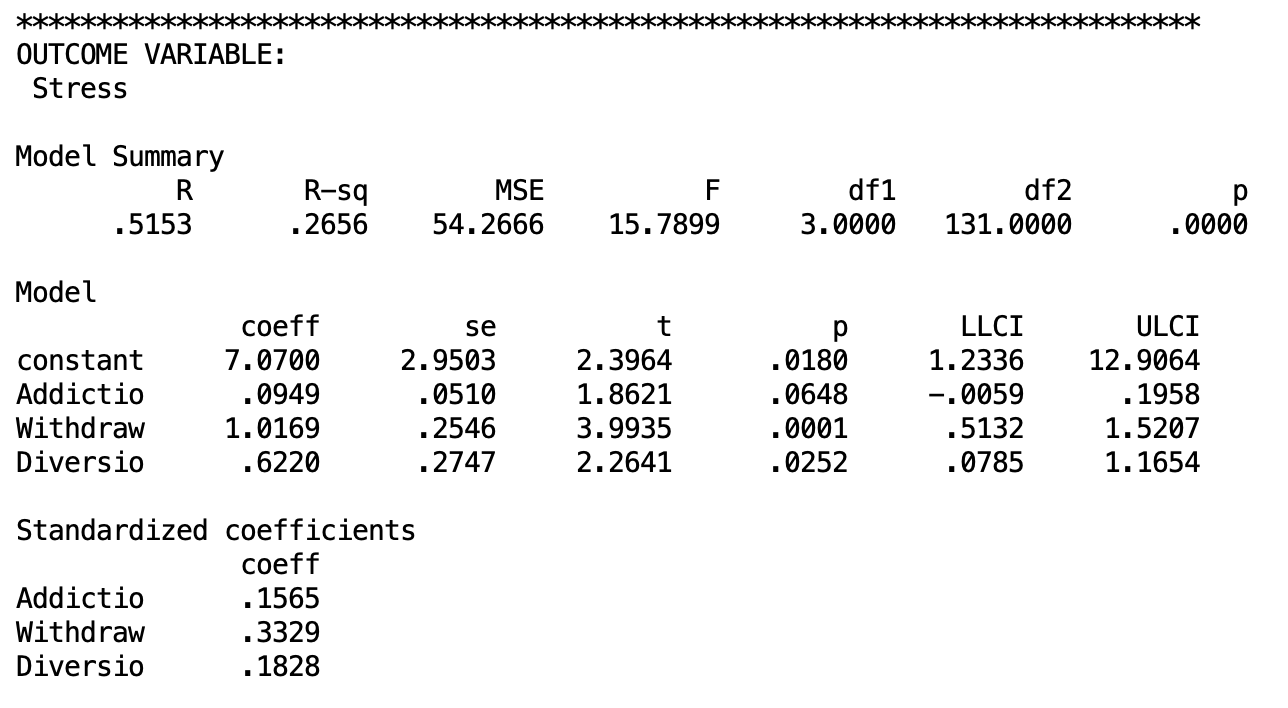


**Model 4**

Engagement

Depression

Diversion

Withdrawal

Approach


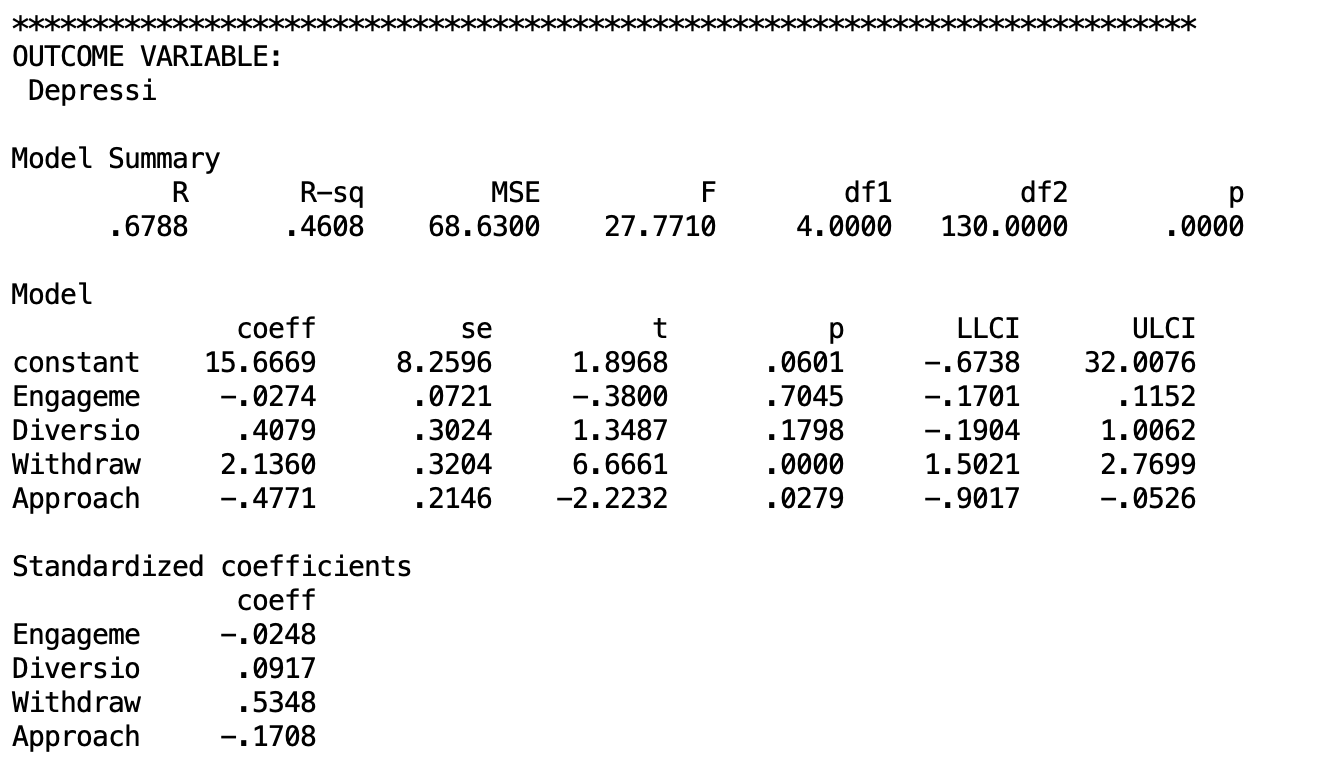


**Model 5**

Engagement

Anxiety

Diversion

Withdrawal

Approach


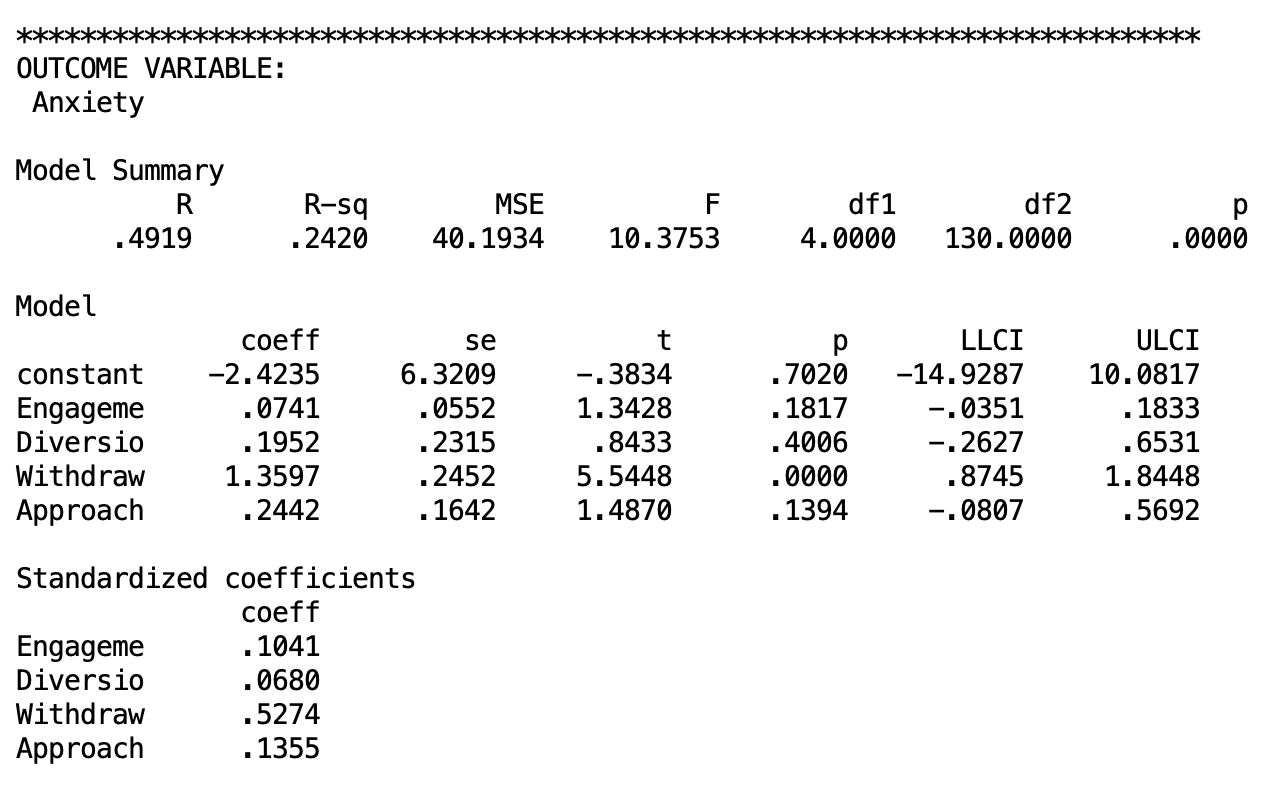


**Model 6**

Engagement

Stress

Diversion

Withdrawal

Approach


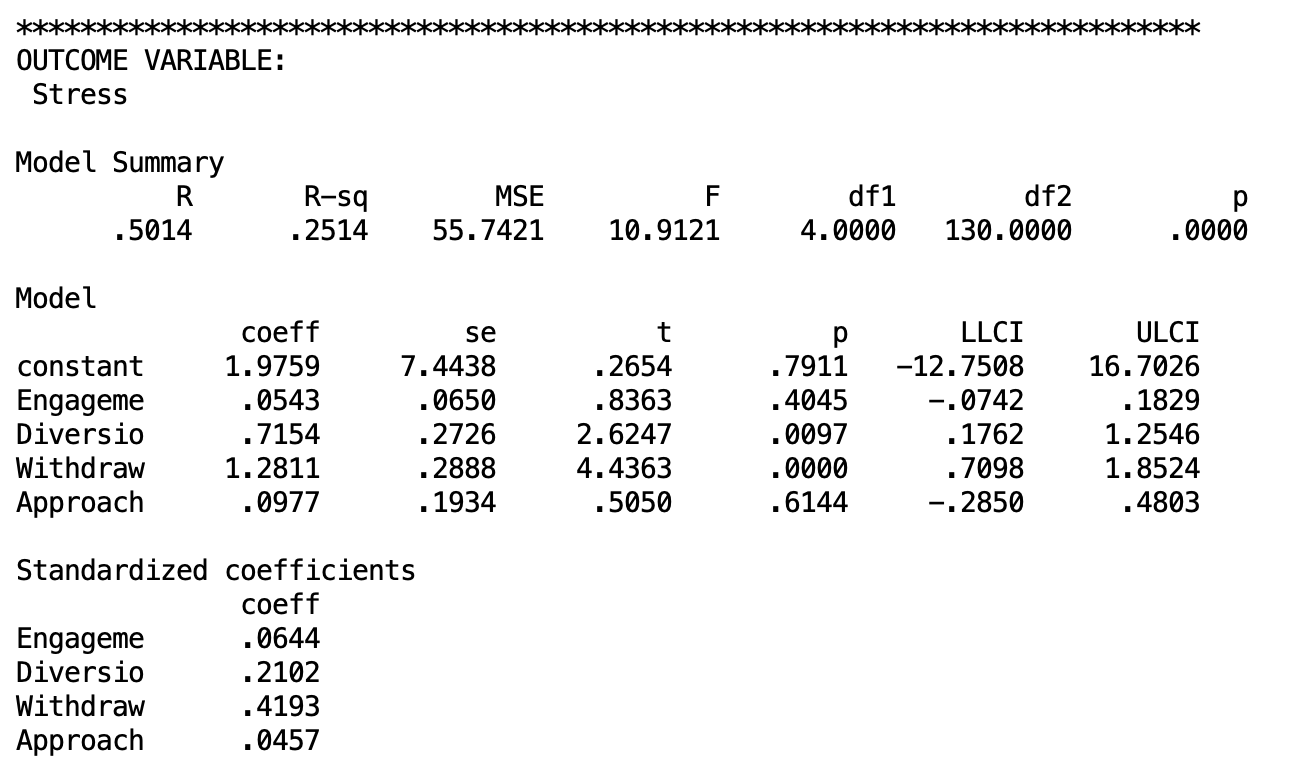


**Model 7** (corresponding to Figure 5 in text)

Addiction

Depression

Offline PSS

Online PSS


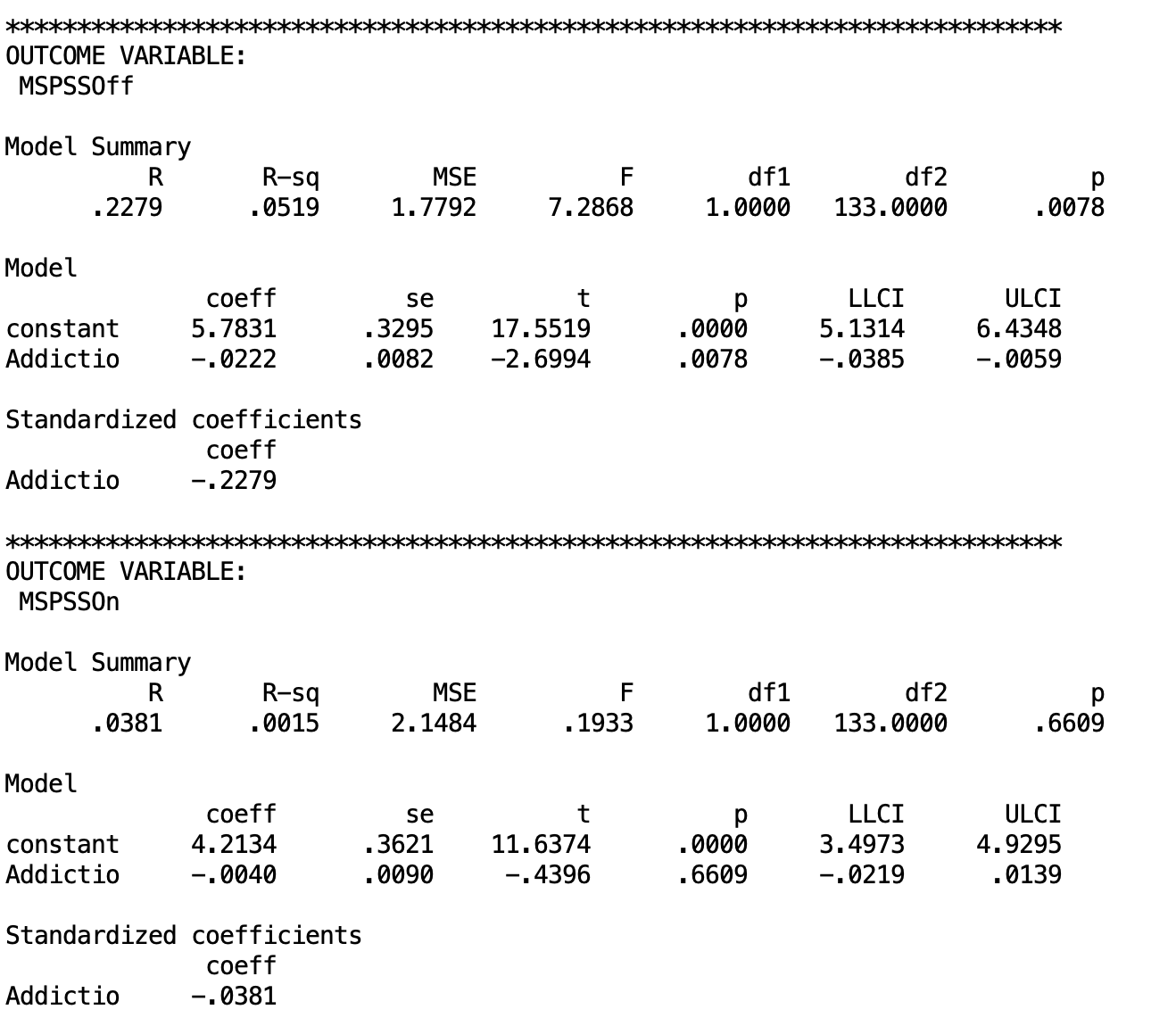


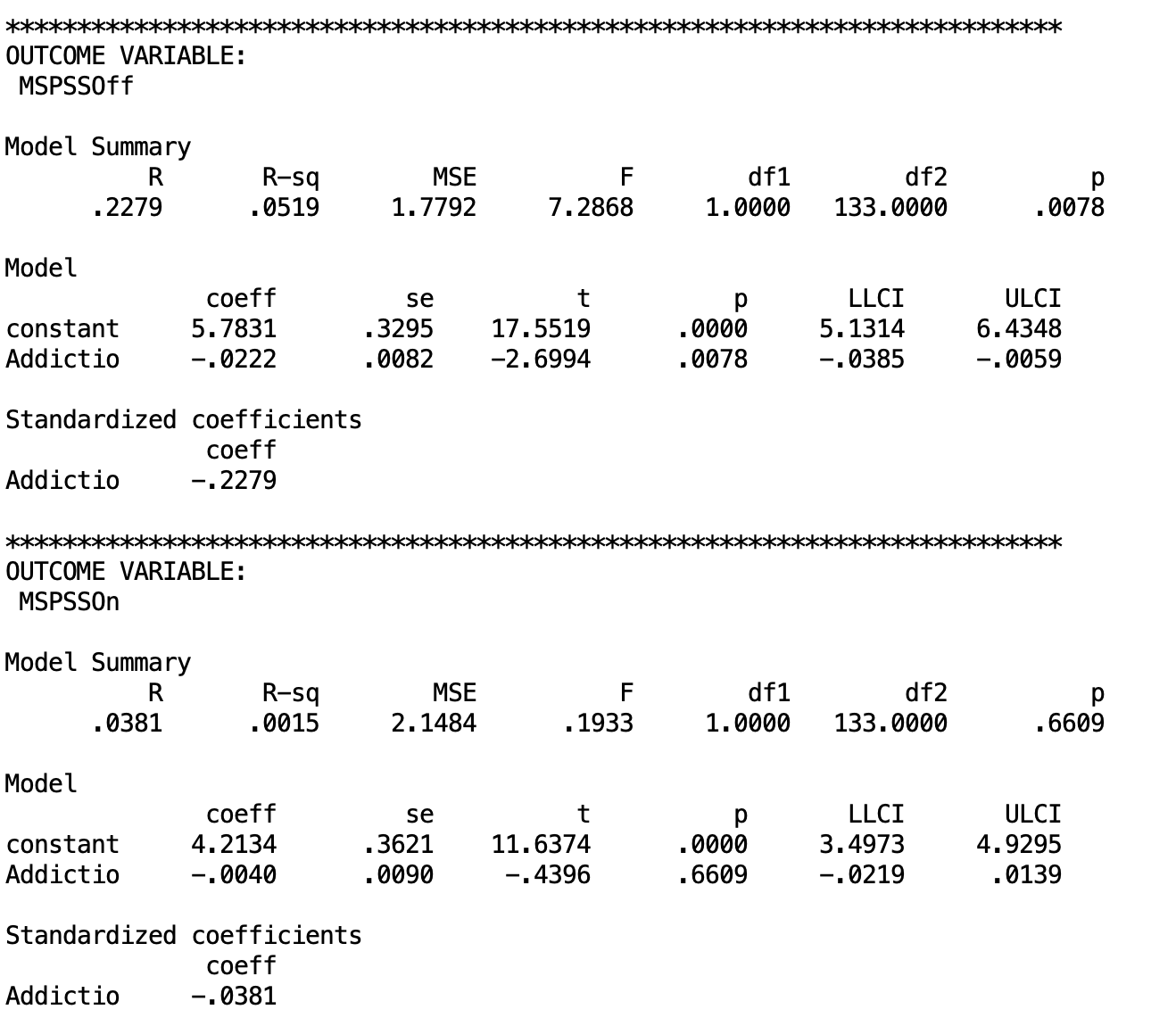


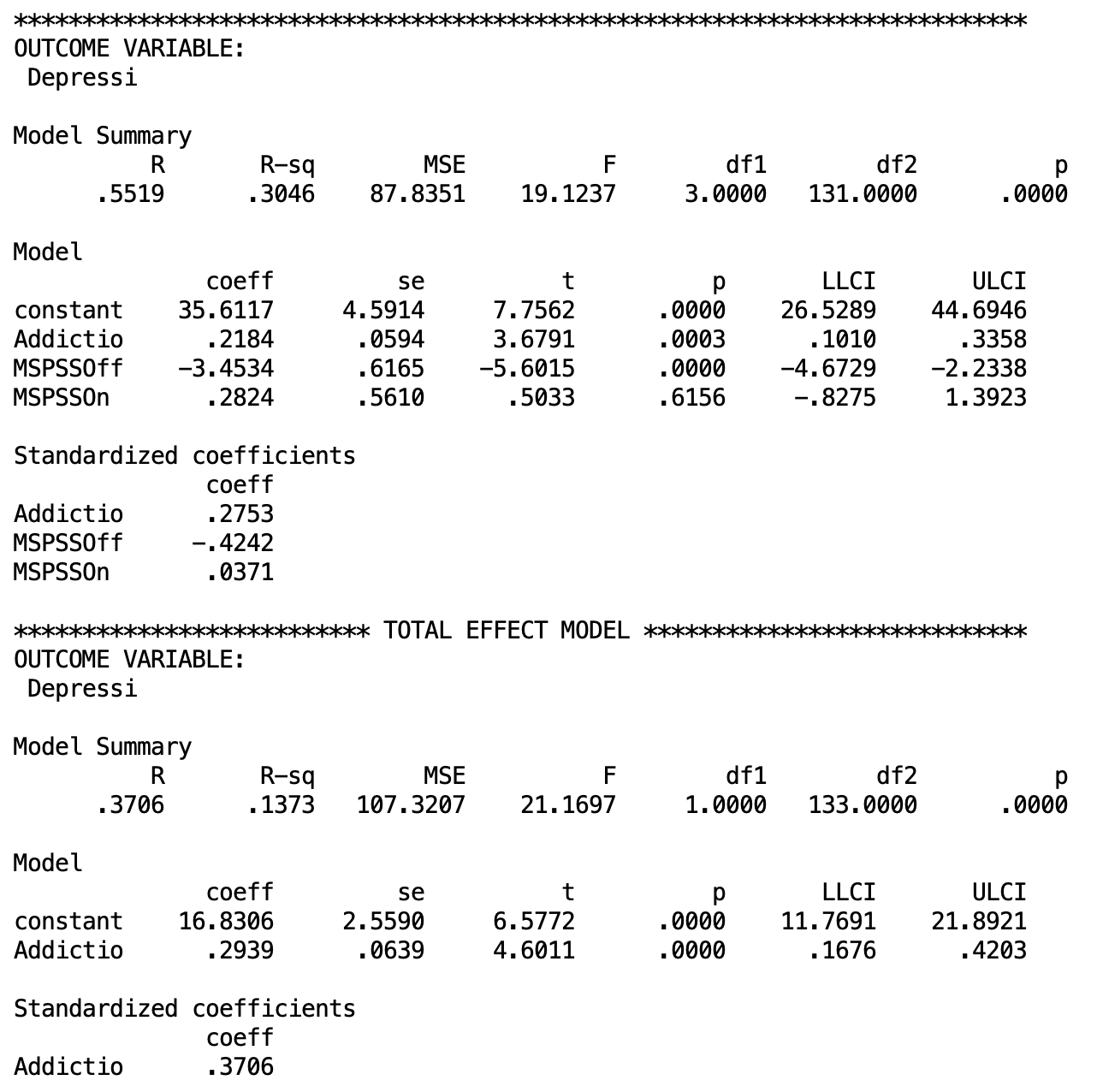


**Model 8** (corresponding to Figure 6 in text)

Addiction

Anxiety

Offline PSS

Online PSS


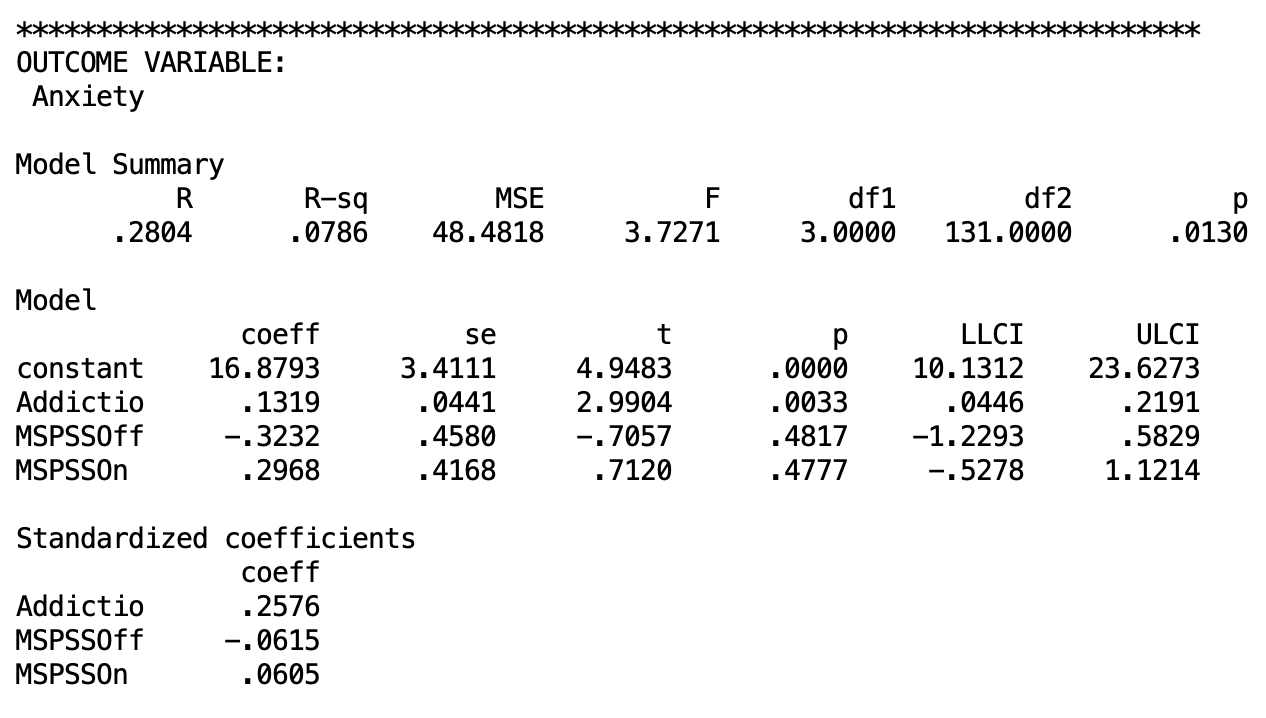


**Model 9** (corresponding to Figure 7 in text)

Addiction

Stress

Offline PSS

Online PSS


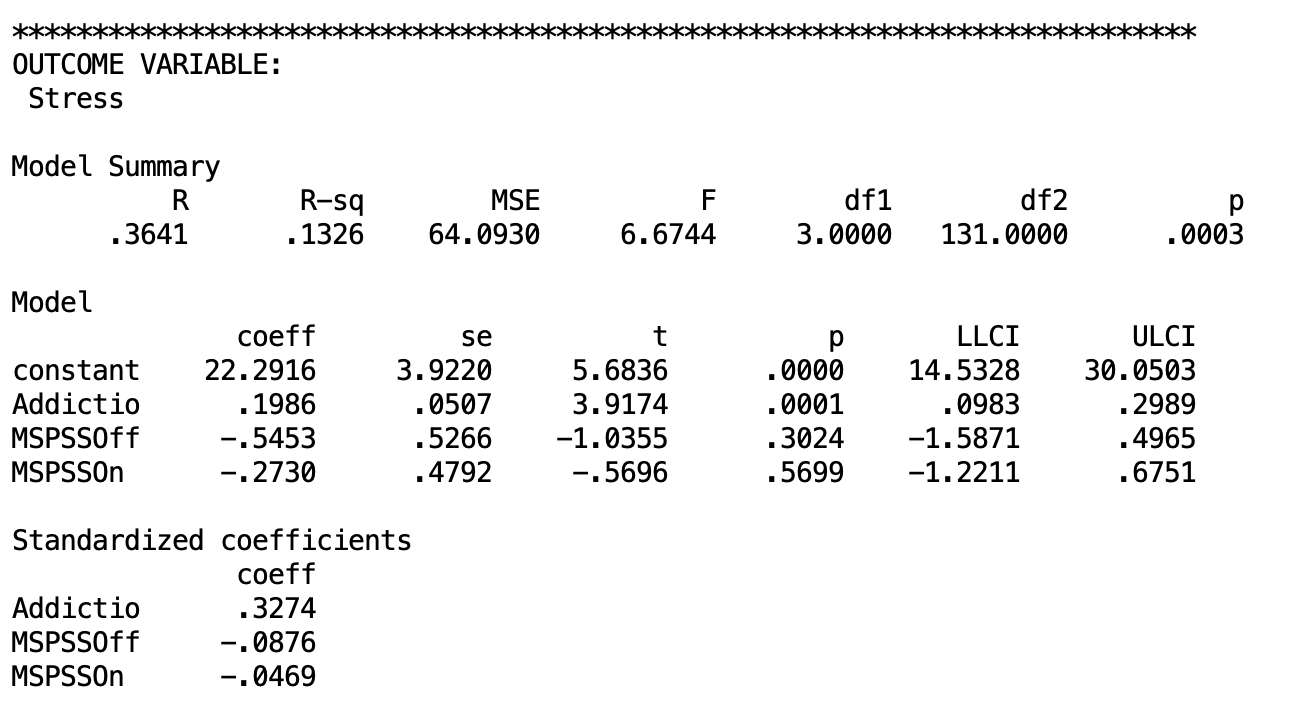


**Model 10**

Engagement

Depression

Offline PSS

Online PSS


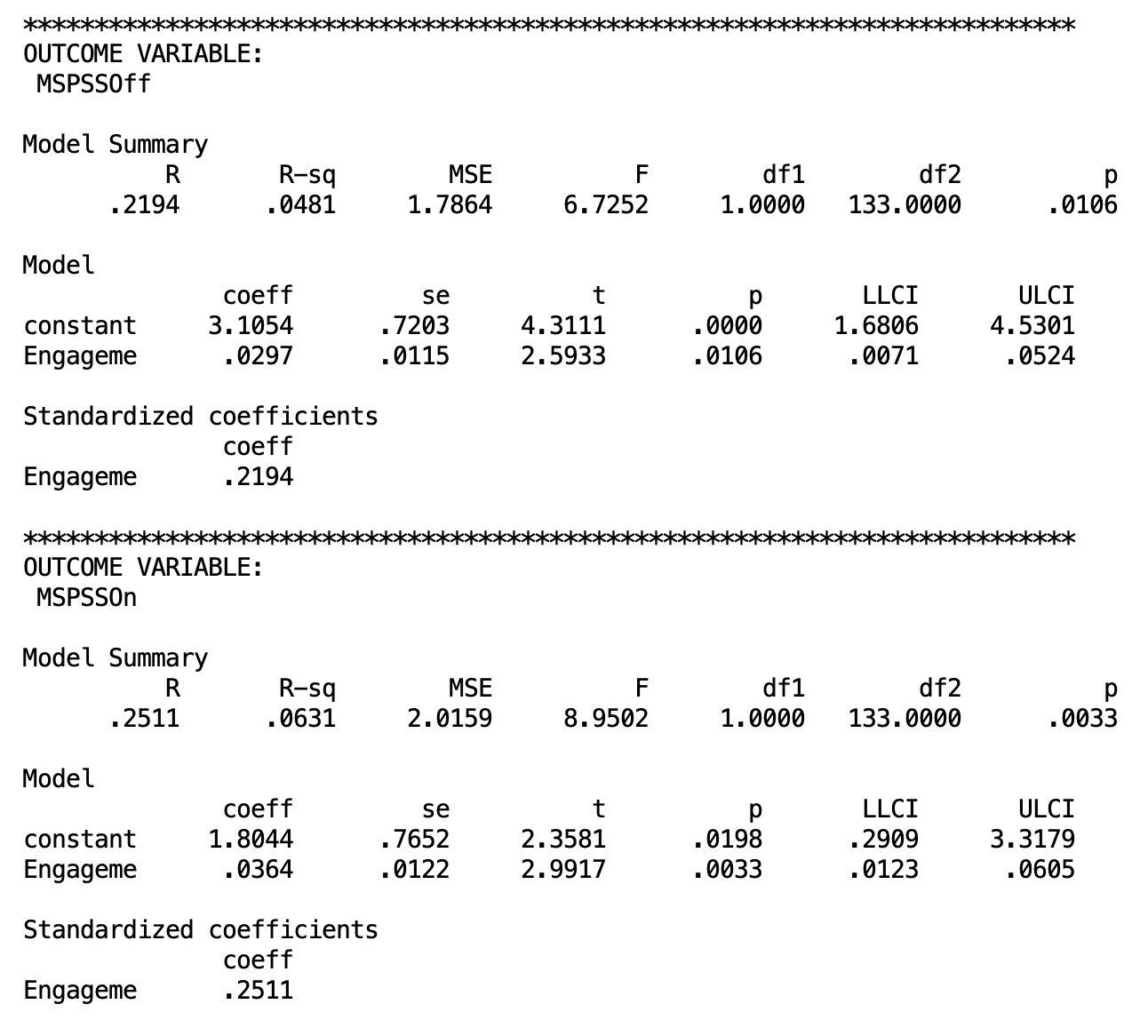


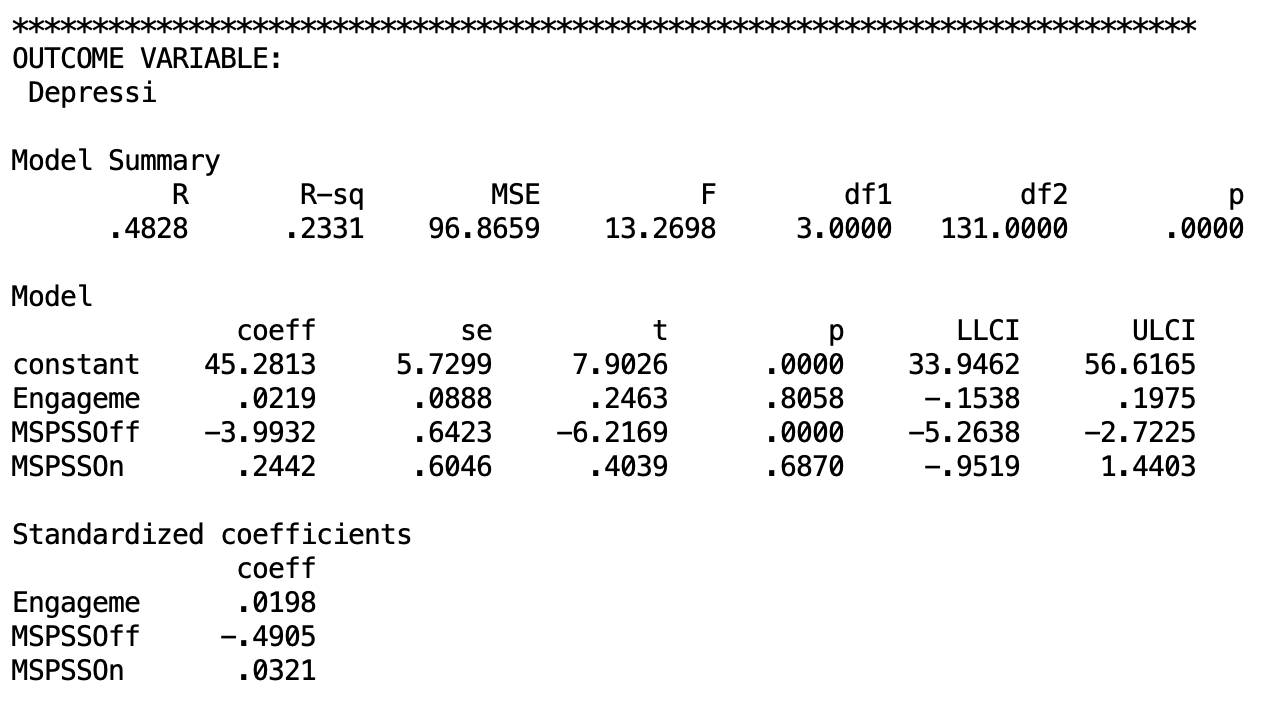


**Model 11**

Engagement

Anxiety

Offline PSS

Online PSS


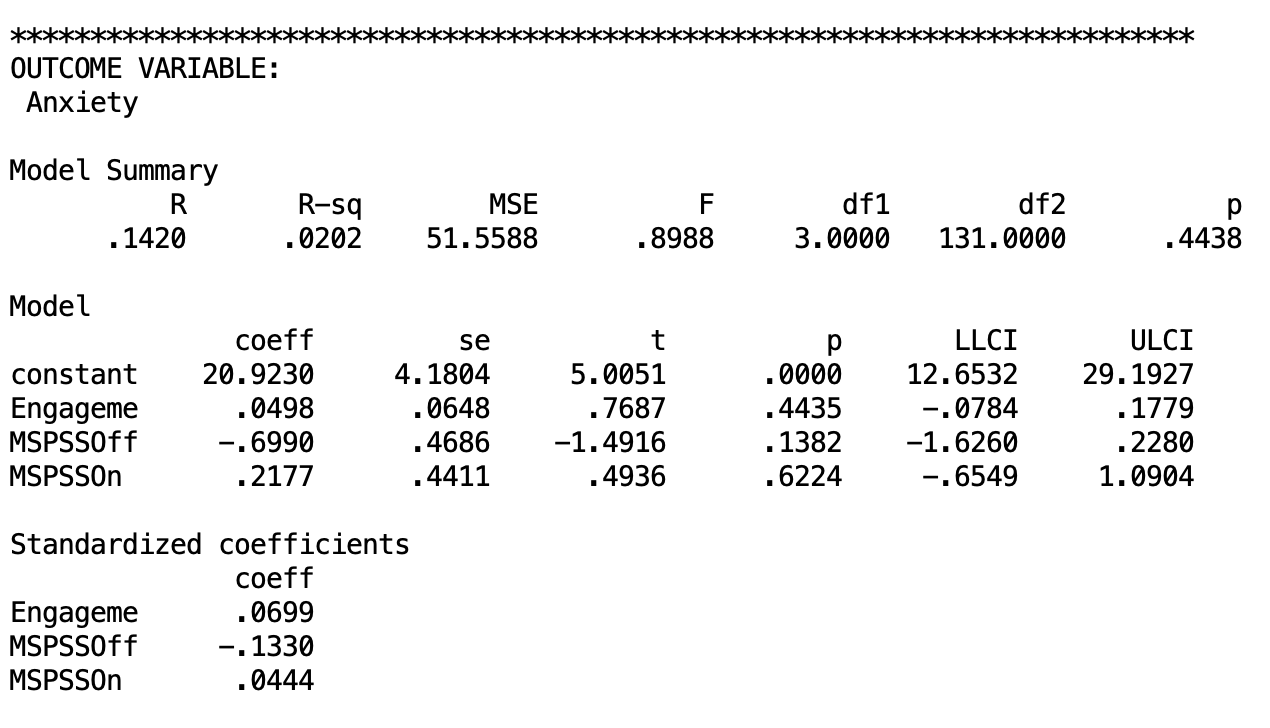


**Model 12**

Engagement

Stress

Offline PSS

Online PSS


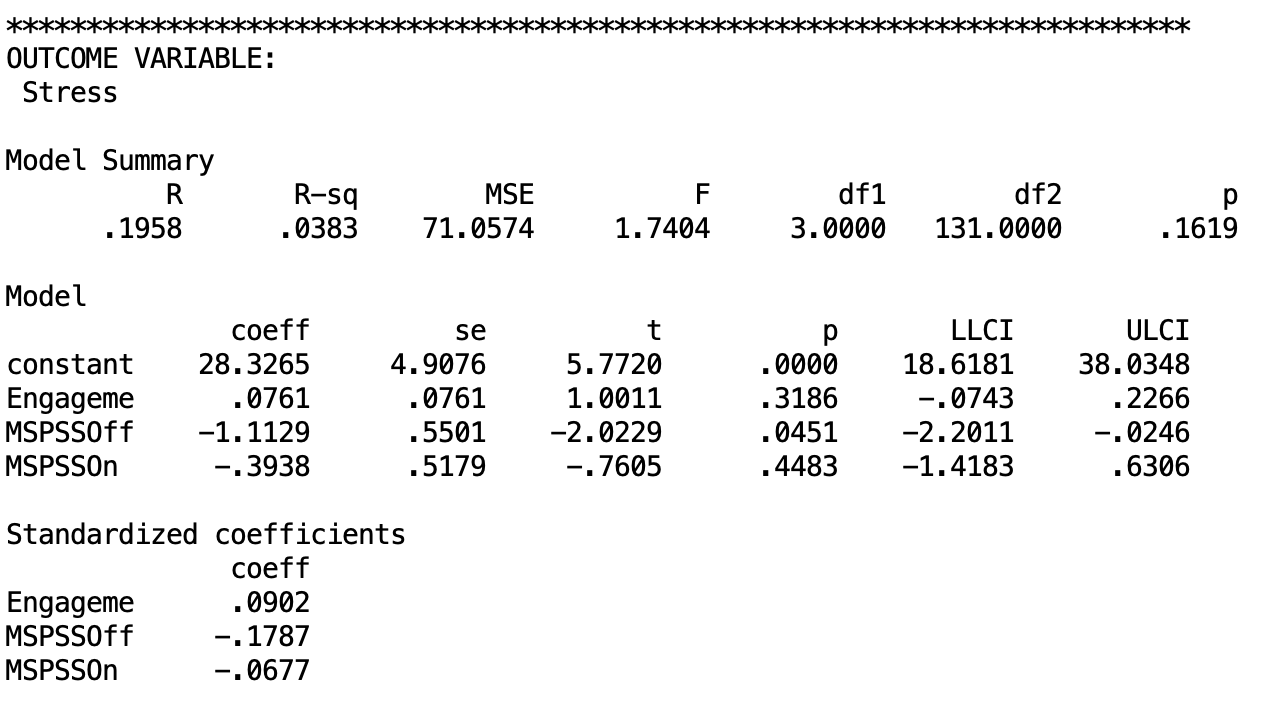

Supplement: Heliyon Supplementary Material - Appendices.docx [file mmc1.docx]
